# The effect of coronal pre-flaring and root canal irrigant on apex locators’ accuracy: an in-vitro study

**DOI:** 10.12688/f1000research.133288.1

**Published:** 2023-05-23

**Authors:** Shimaa Rifaat, Abdullah Aljami, Turki Alshehri, Shahad T. Alameer, Alhanoof Aldossary, Wejdan Almutairi, Mulham N. Almaliki, Faraz A. Farooqi, Noha Taymour

**Affiliations:** 1 Department of Restorative Dental Sciences, College of Dentistry, Imam Abdulrahman Bin Faisal University, P.O Box 1982, Dammam, 31411, Saudi Arabia; 2 College of Dentistry, Imam Abdulrahman Bin Faisal University, P.O. Box 1982, Dammam, 31411, Saudi Arabia; 3 Department of Dental Education, College of Dentistry, Imam Abdulrahman Bin Faisal University, P.O. Box 1982, Dammam, 31411, Saudi Arabia; 4 Department of Substitutive Dental Sciences, College of Dentistry, Imam Abdulrahman Bin Faisal University, P.O. Box 1982, Dammam, 31411, Saudi Arabia

**Keywords:** Working length, Root ZX apex locator, Raypex 6 apex locator, irrigating solution, coronal pre-flaring, sodium hypochlorite, chlorhexidine, dry medium.

## Abstract

**Background:** Successful root canal treatment is influenced by the apical extent of root canal preparation and the eventual root canal filling. Achieving the full working length until the apical constriction, which is usually 0.5 – 1 mm shorter than the anatomical apex, is crucial. Electronic apex locators were used to detect the working length more accurately. There are six generations of electronic apex locators in the market. The selection of the appropriate irrigation with each apex locator for accurate working length determination is not fully investigated.

**Methods:** The actual working lengths of 120 freshly extracted human single-rooted teeth were measured and compared with their working lengths using 3 ^rd^ generation (Root ZX) followed by 6 ^th^ generation (Raypex 6) apex locators in dry medium, presence of 5.25% sodium hypochlorite, and 2% chlorhexidine, without coronal pre-flaring and after coronal pre-flaring using the same irrigating media. Data were collected, tabulated, and afterward analyzed using one-way ANOVA with post-hoc to evaluate the significant difference in average working length between actual working length, Root ZX, and Raypex 6 apex locator working lengths accuracy.

**Results:** The significant results were shown in roots that were coronally pre-flared and their working lengths were measured in a dry medium using Raypex 6 apex locator. While using the Root ZX apex locator, the most accurate results were shown in roots that were coronally pre-flared and their working lengths were measured while using a chlorhexidine irrigating solution.

**Conclusions:** It is concluded that it is very important to know the specific irrigating medium to be used with each specific electronic apex locator to achieve the most accurate working length results.

## Introduction

Successful root canal treatment (RCT) is influenced by the apical extent of root canal preparation and the eventual root canal filling. ^1^ Because the canal is narrow at the apical constriction, it is the suggested endpoint for instrumentation and obturation. ^2^ Apical constriction is defined as the minor root canal diameter. Histologically, it represents the transitional point between the pulpal and the periodontal tissue at the cement-dentinal junction (CDJ). According to anatomical research, the apical constriction is 0.5 – 1.0 mm from the exterior or the main foramen. ^3^ Therefore, the full working length (WL) until the apical constriction, which is 0.5 – 1 mm shorter than the anatomical apex, provides a clean barrier that protects the periodontium from any bacterial invasion. ^3^ Moreover, under or overfilling of the canals is one of the reasons for endodontics treatment failures. ^4^^–^^6^

Accordingly, working length determination is a critical step in endodontic treatment. ^3^ There are many ways to determine the working length (WL), which are: periapical X-ray (PAs), bleeding point, electronic apex locators, and the tactile sensation of the operator. ^7^ The most commonly used method is to combine the usage of electronic apex locators and periapical radiographs. ^8^ To reduce the patient’s exposure to the X-ray and take the least number of X-rays, a reliable apex locator should be used. There are six generations of electronic apex locators (EAL) used to detect the working length. The most popular system used is the 3 ^rd^ generation apex locators. ^9^ It uses two frequencies and measures the difference between these frequencies; however, the type of moisture present may affect the reading’s accuracy. ^9^^,^^10^ The 4 ^th^ generation, on the other hand, uses five frequencies, but they make mathematical measurements rather than measure according to a database. ^9^^,^^10^ However, there are no marked differences in reading accuracy compared to the older apex locator generations. The 5 ^th^ generation uses a database of canal electric features and then does a comparison along with a mathematical process. The new technological advancement has led to the sixth generation of EAL, where a steady algorithm is made according to the canal’s moisture properties. Furthermore, working length measurements became more reproducible and accurate than in the previous generations. ^9^

Using an irrigating media in determining working length is being studied in the literature. Keeping the root canal dry or moist using an irrigant during using the apex locators has been questioned by many dental practitioners, whether it increases the accuracy rate of EAL or not. Using 5.25% sodium hypochlorite (NaOCl) during root canal treatment meets the prime principle of endodontics, chemo-mechanical cleaning and shaping of the root canal. NaOCl is also used as an antibacterial agent to dissolve organic components of the root canal system. ^11^

2% chlorhexidine gluconate (CHX) is a broad-spectrum antiseptic cationic agent which comes in two forms: gel (CHX-G) and solution (CHX-S). However, it does not have the ability to dissolve organic materials as 5.25% NaOCl does. ^11^ A 2% CHX has been used in endodontics as an irrigating material or intracanal medicament as it has a broad spectrum of antimicrobial activity and a lower cytotoxic effect than NaOCl. While demonstrating its successful clinical performance, lubricating properties, and rheological action (in the gel form), it also prevents metalloproteinase, is chemically stable, does not stain clothes, is odorless, water-soluble, and due to its cationic structure gives a unique feature known as substantivity (residual antimicrobial activity). ^12^^,^^13^ It is well-established that the chemo-mechanical procedure can be enhanced if followed by the usage of an antibacterial intracanal medication like chlorhexidine (CHX), especially in cases of exudation, hemorrhage, perforation, root resorption, trauma, or insufficient root development. ^13^ Coronal pre-flaring for root canals has several advantages that can be reflected on the cleaning and shaping processes, including making it easier to put manual and rotary equipment into the apical region of the root canals. ^14^^,^^15^ The results indicated that the pre-flaring procedure provided more accurate measurements in most cases. ^16^

Few studies had been conducted to evaluate the effect of irrigating solutions on the working length reading accuracy of various electronic apex locators in the presence or absence of coronal pre-flaring. Hence, this present study aimed to compare the working length (WL) accuracy using the 3 ^rd^ generation (Root ZX) and 6 ^th^ generation (Raypex 6) electronic apex locators (EAL) in single-rooted teeth in the presence of different irrigation media: dry medium, 5.25% sodium hypochlorite (NaOCl), and 2% chlorhexidine (CHX) in root canals with/without coronal pre-flaring.

## Methods

### Ethical approval

The present study was conducted in compliance with the Declaration of Helsinki, and the research protocol was approved by the Restorative Dental Science Department, Imam Abdulrahman Bin Faisal University, College of Dentistry. Ethical approval was obtained from the Institutional Review Board, at Imam Abdulrahman Bin Faisal University (IRB -2022-02-171) on 12/04/2022.

Teeth were collected in disposal after receiving signed consent from dental surgery/oral procedure patients, who authorized the hospital to use its discretion in their disposal and to be used in research purposes if needed.

### Sample size calculation

Power analysis was performed using the Clinclac sample size for this study. Mean and standard deviations were used from previously published literature ^11^ which are (0.64±0.54) and (0.33±0.22). The power of the sample was set as 90% and the significance level will be set as 0.05. Hence the calculated total sample size obtained was 109 and the sample was increased to 120 for more precise results.

### Sample selection

This research began on 17/04/2022. 120 freshly extracted human single-rooted teeth were used in this study. Teeth were collected from the disposal section at Imam Abdulrahman Bin Faisal University Hospital after receiving signed consent from dental surgery/oral procedure patients, who authorized the hospital to use its discretion in their disposal and to be used in research purposes if needed. The extracted teeth were collected anonymously without exposing any of the patient’s data and were used only for this in-vitro study. They were collected and immersed in 5.25% sodium hypochlorite (NaOCl) for disinfection for 2 hours for disinfection. ^3^ Afterward, they were stored in normal saline until their usage. Root surfaces and the apical regions’ examinations were done under a dental operating microscope (OMS1950 Dental Microscope, USA) with 25X magnification. Teeth with comparable lengths and completely formed apices were included in the current study. Teeth with any possible fractures and/or apex immaturity were excluded from the current study. Teeth were radiographed in both mesiodistal and buccolingual directions to exclude the absence of root resorption or canal curvatures. Only root canals with a curvature of 0–5 degrees were included in the study. Teeth with calcified canals, more than one canal, apical blockage, internal or external resorption, and caries all were excluded from this study.

### Sample preparation

120 freshly extracted human single-rooted teeth were used for this study. They were examined using periapical X-ray for 0.08 seconds of exposure time. Scaling of the teeth was performed using an ultrasonic scaler (Dentsply Sirona, ProUltra Piezo Ultrasonic Handpiece), then stored in normal saline at 0.9% until used. Teeth were flattened with a diamond disk 1 mm thickness (Hi-Tech diamond disc bur) to have a reliable reference point based on Jakobson *et al.* findings that the rubber stopper on the file should be placed on a flat surface to limit the possibility of errors in research with electronic apex locators, which ensured that the study’s findings are not influenced. ^17^ The working length was evaluated by two evaluators who were instructed to use the same criteria for evaluating and assessing the parameter of the current study. Cohen’s Kappa test was applied to ensure the agreement and consistency between the two evaluators’ WL evaluations. A value of 0.82, which is interpreted as a high level of agreement or reliability between the two evaluators, was gained.

### Working length measurements without coronal pre-flaring

Conventional access opening without any coronal pre-flaring was done. Apical patency was checked using K-file #10 (Dentsply M-access K-File). Actual working length (WL) was measured in millimeters by two calibrated evaluators under an endodontic microscope (OMS1950 Dental Microscope) with 25× magnification. Any tooth with an initial file more than #15 K-File (Dentsply M-access K-File) was excluded. The file was placed beyond the apical constriction and then retrieved until it is flushed with apical foramen. A 0.5 mm was subtracted from the total and the final measurement was considered the actual working length. ^2^^,^^8^ Each tooth was mounted until the cementoenamel junction (CEJ) level using freshly mixed alginate. A double rubber stopper technique was used. ^3^

### Working length determination

The working length in millimeters for each tooth was measured using (3rd generation EAL) Root ZX (J. Morita Corp., Kyoto, Japan) then followed by using (6 ^th^ generation EAL) Raypex 6. (VDW, Munich, Germany). Apex locators were used while teeth were dry, then while using 5.25% NaOCl, then while using 2% CHX respectively. Between each irrigating solution and the next, distilled water was used to neutralize each irrigant effect before using the next one. Afterward, the canals were dried using paper points. All measurements were taken by two calibrated examiners. K-file size #15 (Dentsply M-access K-File) was inserted inside the canal, a lip clip was placed inside a fresh alginate mix and a file holder was placed on the file.

The file was fixed on apex locators: the third generation EAL Root ZX (J. Morita Corp., Kyoto, Japan) and the sixth generation EAL Raypex 6 (VDW, Munich, Germany) for 5 seconds before measurements were recorded. Measurements were recorded when the file reached the mid-green area on the EALs’ screens. ^18^

### Working length measurements with coronal pre-flaring

**Figure 1.**
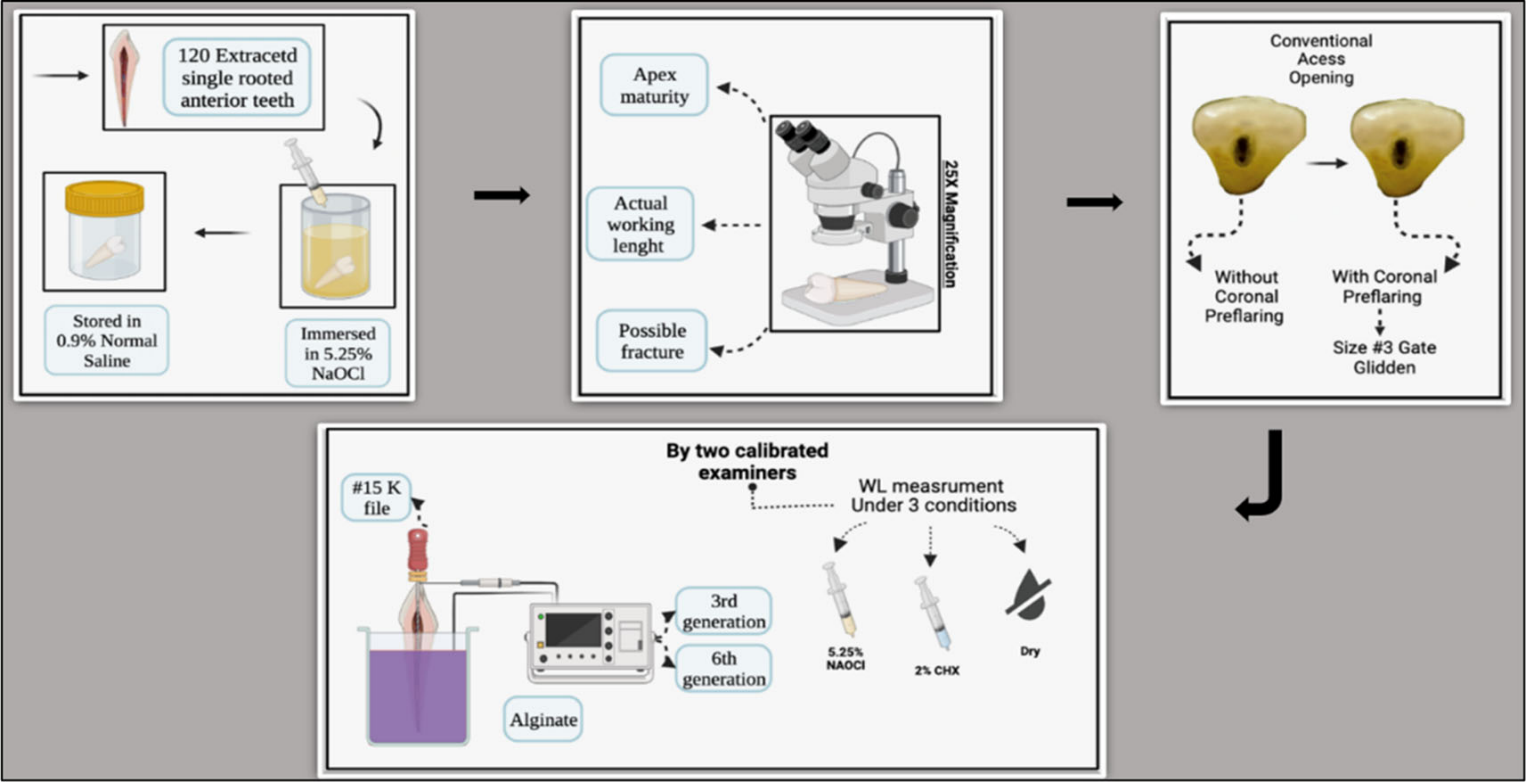
Schematic drawing showing the followed steps for the study from teeth selection, teeth storage, measuring the actual working length under the microscope, access opening preparation without coronal pre-flaring followed by access opening preparation with coronal pre-flaring, teeth mounted in alginate, holding the file in double stopper technique, measuring the working length (WL) in millimeters using different irrigation media (dry medium, 5.25% NaOCl, 2% CHX) with 3 ^rd^ and 6 ^th^ generations electronic apex locators.

All teeth were collected, and coronal pre-flaring of the root canals was done. An access opening was prepared for each tooth. The penetration depth of gates-glidden drills was as follows: #3 to the canal orifice, #2 to the coronal third, and maximum to the coronal half of the canal to avoid perforations and to achieve a straight line access. ^19^ Measuring the working length (WL) in millimeters of all teeth was repeated after the coronal pre-flaring with both Root ZX (3 ^rd^ generation) and Raypex 6 (6 ^th^ generation) apex locators using the dry medium, 5.25% NaOCl, 2% CHX irrigating solutions again as in Figure 1.

### Statistical analysis

Data was initially recorded in an excel sheet and then transferred to SPSS (statistical package for social sciences) version 24, IBM, Inc. Mean and standard deviations were calculated and presented in tables as part of descriptive statistics. Comparisons between the irrigant solutions for Root ZX and Raypex 6 EAL were calculated and tested using the independent sample t-test and ANOVA. Where ANOVA was significant, multiple comparisons were done using Tukey’s post hoc test. Comparison in the working length between the 3 ^rd^ (Root ZX) and 6 ^th^ generation (Raypex 6) apex locators in teeth without coronal pre-flaring and with coronal pre-flaring in all media was also done using an independent sample t-test. P-values less than or equal to 0.05 were considered statistically significant.

## Results

**Figure 2.**
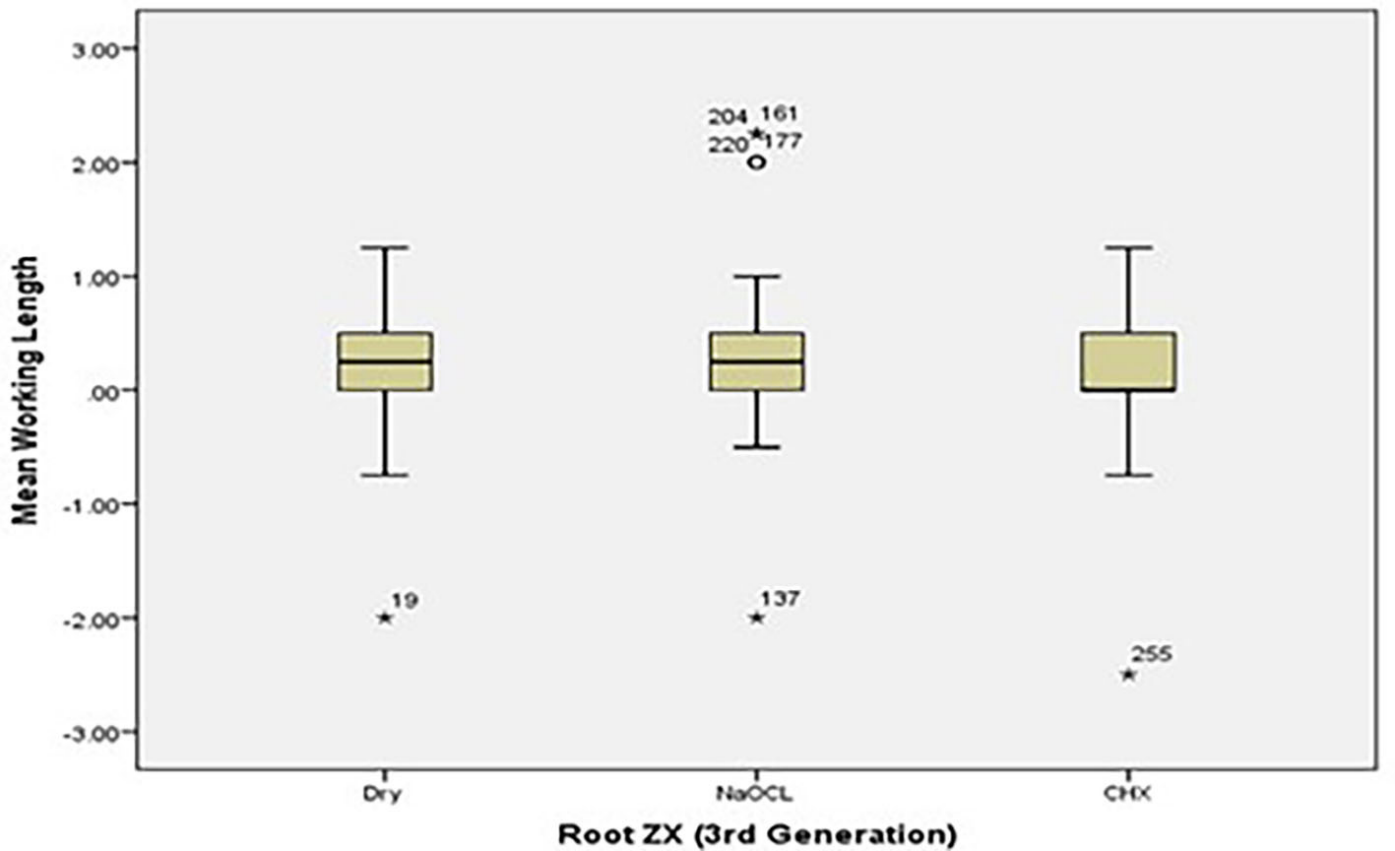
Working length measurements for Root ZX for all irrigating solutions without canal pre-flaring.

**Figure 3.**
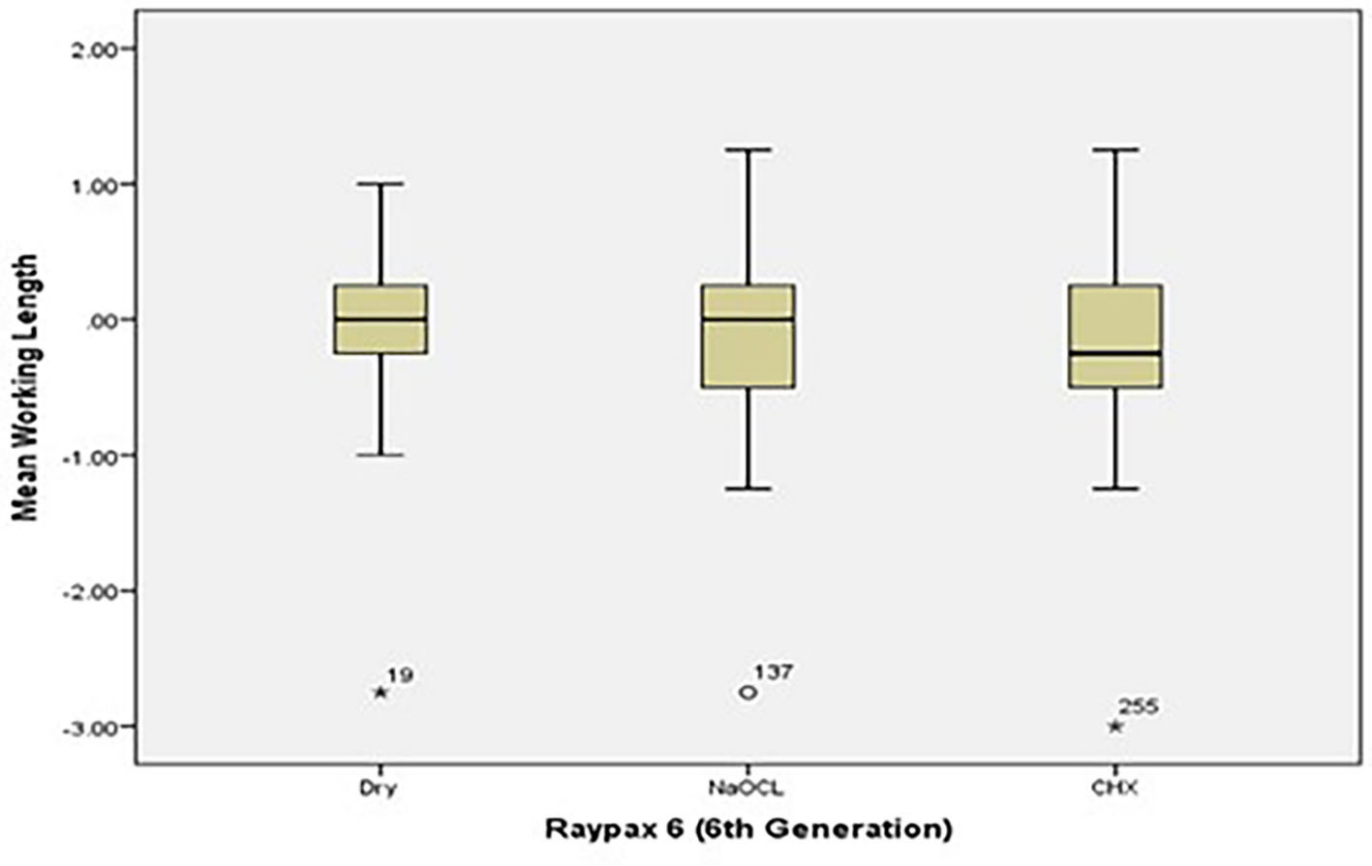
Working length measurements for Raypex 6 for all irrigating solutions without coronal pre-flaring.

Table 1 presents the comparisons of the mean working length (WL) of Root ZX and Raypex 6 EALs between the irrigating solutions and within the irrigating solutions in the absence of coronal pre-flaring. Mean length measurements for Root ZX in CHX were significantly closer to the actual WL (0.087±0.445) compared to the other two irrigants; the least close measurement from the WL was with NaOCl (0.252±0.553), and the difference was statistically significant (p=0.025). Whereas with Raypex 6, the dry medium should have the closest readings to the WL but the overall mean difference among the irrigants used did not show any significant difference. Within each irrigating solution, the mean length of Raypex 6 was close to the actual WL and found to be statistically significant. Figures 2 and 3 present the pre-flaring working length measurements for Root ZX and Raypex 6 for all irrigating solutions. The median lengths for dry medium and NaOCl are almost the same, whereas the median length for CHX significantly decreased and was closer to the actual WL; asterisks are used to show the outliers.

**Table 1.** Comparison of mean working length (WL) of Root ZX and Raypex 6 EALs in the presence of Dry medium, 5.25% NaOCl, 2% CHX media without coronal pre-flaring.

	Without pre-flaring			P-values ANOVA
	Dry	NaOCl	CHX	
**Root ZX (3** ^**rd**^ **generation)**	0.146±0.399	0.252±0.553 ^a^	0.087±0.445 ^a^	F=3.7, P=0.025 *
**Raypex 6 (6** ^**th**^ **generation)**	-0.074±0.486	-0.121±0.506	-0.161±0.534	F=0.861, P=0.42
**P-values T-test**	T=3.8, P=0.001 *	T=5.4, P=0.001 *	T=3.85, P=0.001 *	

* Statistically significant at 0.05.

^a^ Same alphabet showing significant difference between solutions.

**Figure 4.**
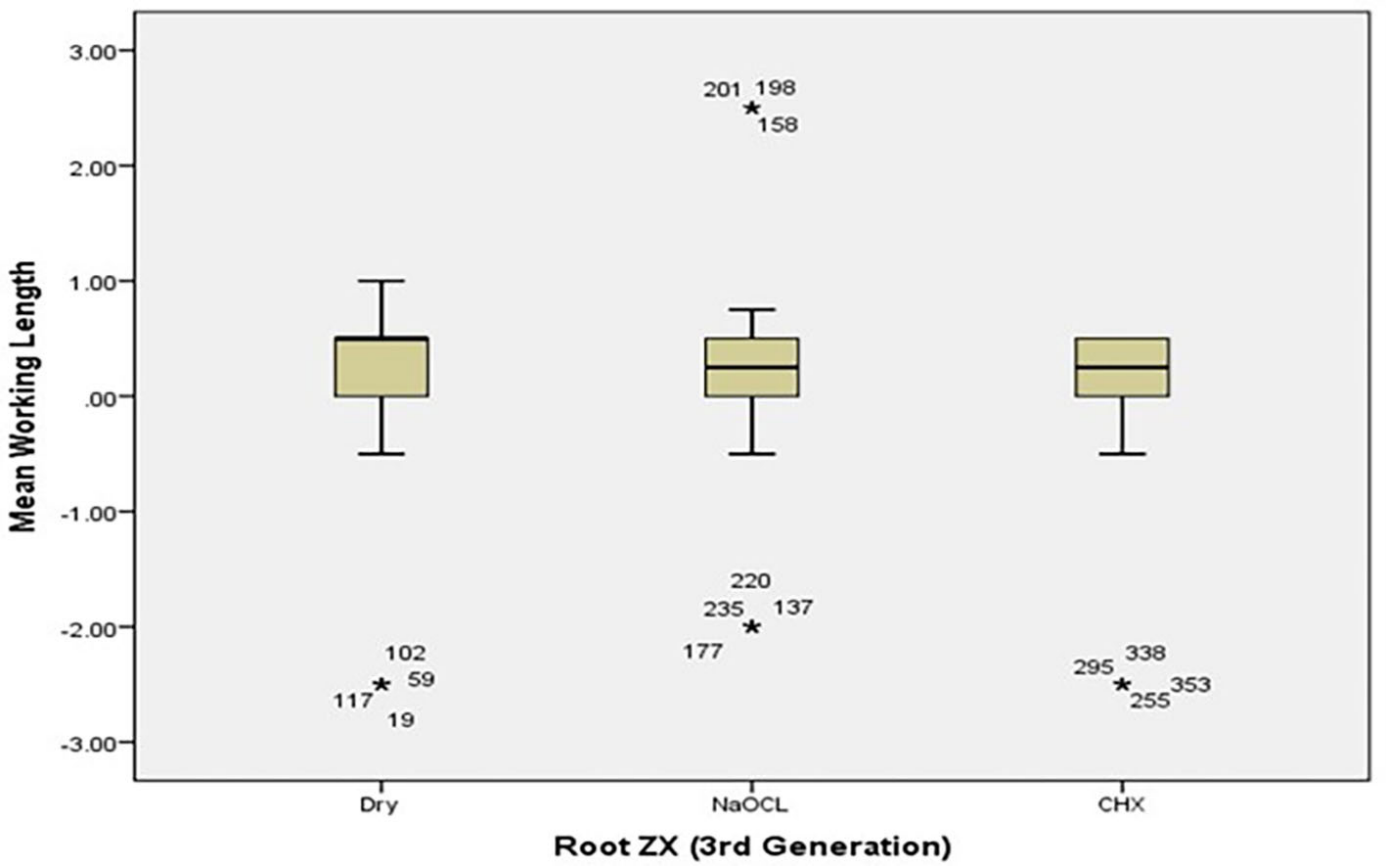
Working length measurements for Root ZX for all irrigating solutions with coronal pre-flaring.

**Figure 5.**
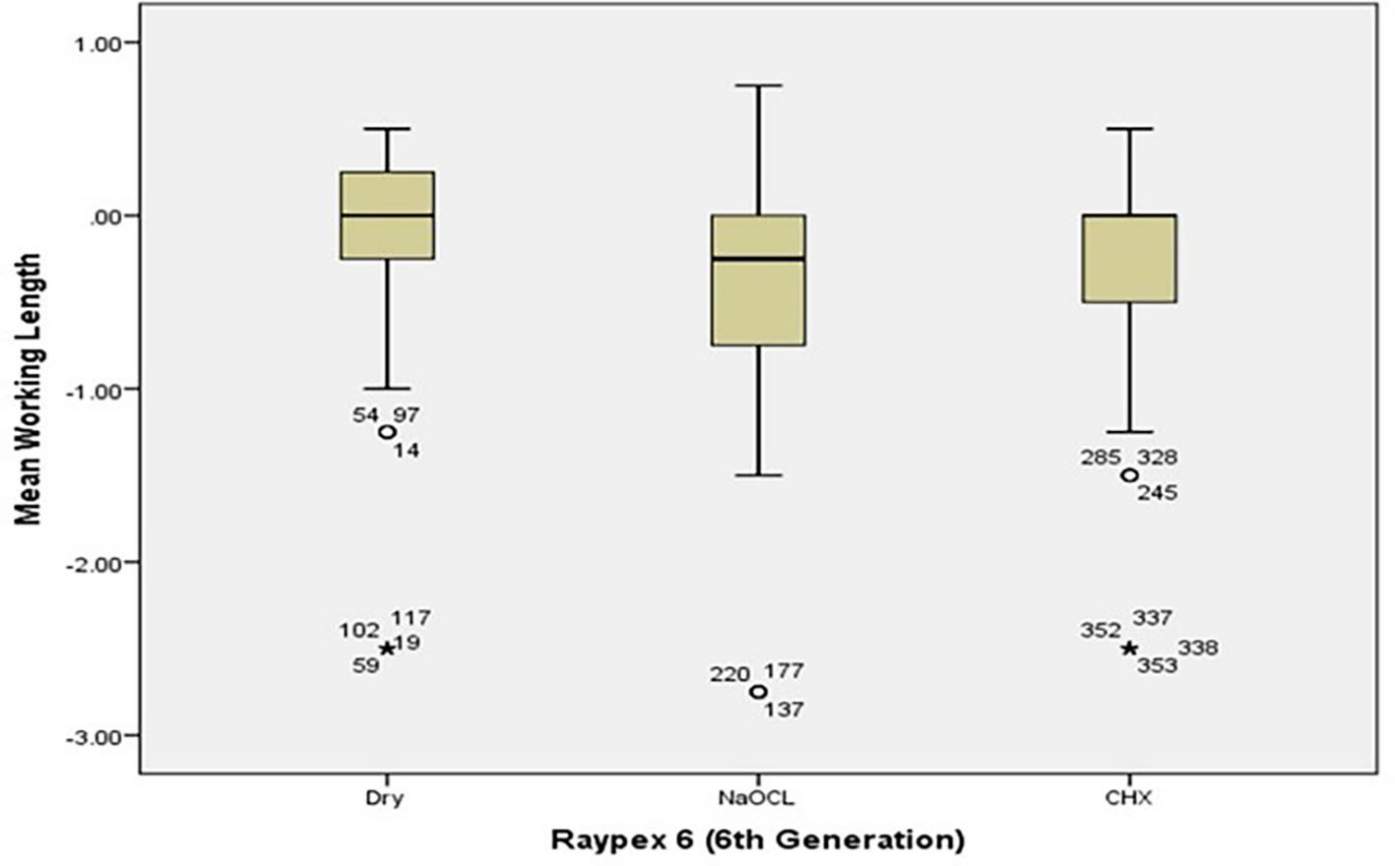
Working length measurements for Raypex 6 for all irrigating solutions with coronal pre-flaring.

Similarly, Table 2 presents the mean WL difference among the solutions for both apex locators in the presence of coronal pre-flaring. The mean WL for Root ZX locator differs significantly among the solutions (p-0.038). The closest mean to the actual WL was recorded with CHX (0.068±0.586), whereas the mean that most differed from the actual WL was recorded in the dry medium (0.269±0.621). Likewise, the most accurate mean WL for Raypax 6 locator was recorded in dry medium (-0.464±0.641); the least accurate WL was recorded with NaOCl (-0.174±0.584), and the difference between the actual WL and the mean was statistically significant. Within the irrigating solution groups, both apex locators differ significantly Table 2. The box plot in Figures 4 and 5 show the measurements’ median WL spread. Figure 4 shows that the median WL in NaOCl and CHX medium was almost equal but significantly different in the dry medium. Figure 5 shows a similar pattern for Raypax 6 apex locator where asterisks in each box plot refer to the presence of outliers.

**Table 2.** Comparison of mean working length (WL) of Root ZX and Raypex 6 EALs in the presence of Dry medium, 5.25 % NaOCl, 2 % CHX media with coronal pre-flaring.

	With pre-flaring			P-values ANOVA
	Dry	NaOCl	CHX	
**Root ZX (3** ^**rd**^ **generation)**	0.269±0.621 ^a^	0.206±0.64	0.068±0.586 ^a^	F=3.28, P=0.038 *
**Raypex 6 (6** ^**th**^ **generation)**	-0.174±0.584 ^a^	-0.464±0.641 ^a^	-0.28±0.74	F=5.87, P=0.003 *
**P-values**	T=5.4, P=0.001 *	T=8.02, P=0.001 *	T=3.99, P=0.001 *	

* Statistically significant at 0.05.

^a^ Same alphabet showing significant difference between solutions.

The working length for both apex locators was then compared between the presence and absence of coronal pre-flaring and presented in Table 3. Mean length on both occasions (with and without coronal pre-flaring) did not differ significantly for both apex locators in dry medium (p-0.072) but the closest mean WL with actual WL was recorded in without coronal pre-flaring irrigation groups (0.146±0.39, -0.074±0.48, respectively). Similarly, in the NaOCl solution the most accurate mean WL was recorded without coronal pre-flaring media for Raypex 6 apex locator (0.121±0.50) and the difference was statistically significant (p-0.001). The closest mean WL to the actual WL in CHX for Root ZX was found in pre-flaring media (0.068±0.58) whereas for Raypax 6, it was the closest in the same media (0.28±0.74) but the difference was not significant statistically. When compared regardless of irrigation solution the most accurate mean WL to the actual WL was recorded in without coronal pre-flaring media groups for both apex locators (0.161+0.37, -0.118±0.44 respectively) and the difference was only significant statistically for Raypex 6 apex locator (p-0.005).

**Table 3.** Comparison of working lengths of the roots irrigated with different irrigating media with all the flaring conditions (with/without coronal pre-flaring).

Groups	Dry		NaOCl		CHX		Overall	
	Root ZX	Root ZX	Root ZX	Root ZX	Root ZX	Root ZX	Root ZX	Root ZX
**Without pre-flaring**	0.146±0.39	-0.074±0.48	0.252±0.55	-0.121±0.50	0.087±0.44	-0.161±0.53	0.161+0.37	-0.118±0.44
**With pre-flaring**	0.269±0.62	-0.174±0.58	0.206±0.64	-0.464±0.64	0.068±0.58	-0.28±0.74	0.181±0.54	-0.30±0.57
**P-value**	T=-1.807, p=0.072	T=-1.407, p=0.156	T=0.598, p=0.550	T=4.5, p=0.0001 *	T=0.281, p=0.779	T=1.41, p=0.159	T=3.14, p=0.734	T=2.86, p=0.005 *

* Statistically significant at 0.05.

^a^ Same alphabet showing significant difference between solutions.

## Discussion

The establishment of accurate working length (WL) is a crucial step during any root canal treatment; particularly in cases of anatomical limitations, it is useful when used with radiographs to ensure proper determination of the canal working length. ^20^ The coronal pre-flaring of root canals gives many advantages during the meticulous cleaning and shaping procedures, such as facilitating the insertion of either manual and/or rotary files into the apical third of the root canals by removing cervical dentin interferences. ^21^ In addition, the coronal flaring was known to improve the flow of the irrigating solution within the root canal, minimizing the risk of bacterial invasion into the periapical tissue as well as reducing the risk of canal debris and irrigant extrusion during the root canal preparation procedure. ^22^^,^^23^

In recent years, there has been a growing body of evidence that suggests a correlation between the type of root canal irrigating solution used and the success of coronal pre-flaring efficacy. Moreover, the use of root canal irrigants is a major contributor to the success of endodontic treatment. Some studies showed a correlation between the irrigant and the root canal sealer used to ensure proper hermetic seal, ^24^ while others discussed the effect of the proper irrigant on the accuracy of working length determination. ^25^

In the present study, the effect of coronal pre-flaring on the accuracy of actual working length determination was assessed and the results showed that the more consistent, accurate results were observed in the canals that were prepared with coronal pre-flaring before working length (WL) determination in comparison to the canals that were prepared without coronal pre-flaring (see Table 3). These results might be due to the fact that the coronal pre-flaring improves the tactile sensation of the operator in locating the apical constriction. ^26^ In addition, the largest impedance while inserting the file into the root canal is the coronal one-third of the canal. Moreover, coronal pre-flaring reduces file resistance; subsequently, it is easier to insert the file into the canal toward the apex of the root. ^27^

This is in accordance with a previous study that showed that pre-flaring improved the efficiency of the EALs in the mandibular canals and anterior root canals. ^16^ However, it is contradicted by a previous study conducted by João Marcelo da Silva Teixeira *et al.* (2012) who concluded that the usage of Gates Glidden burs for cervical pre-flaring did not significantly influence the accuracy of the apical placement of the apex locator when determining the actual working length due to insufficient removal of coronal dentin when compared with the rotary system for preparing coronal pre-flaring. ^28^

Consequently, the difference was statistically significant in the roots with coronal pre-flaring prior to working length determination when using Raybex 6 apex locator and NaOCl as an irrigant (T=4.5, p=0.0001). While comparing all the groups of Raypex 6 (with or without coronal pre-flaring) regardless of the type of irrigant used, significant results were seen when compared to the same groups of Root ZX (T=2.86, p=0.005).

In the case of using NaOCl as an irrigant, it showed significant results when used with Raypex 6 even without coronal pre-flaring. This coincided with the results of *Elnaghy et al*, *2017* who found that the use of 5.25% sodium hypochlorite (NaOCl) as an irrigant without coronal pre-flaring was associated with greater success rates than 2% chlorhexidine (CHX) solution. This may be due to the good electrical conductivity of NaOCl, which contributes to the accurate detection of Raypex 6 to the apical constriction. Moreover, it may be due to the advanced technology of the Raypex 6 that can accurately work in different canal conditions, including the presence of debris and/or obstruction(s) of the canal. ^9^ Furthermore, the researchers also noted that sodium hypochlorite irrigant was more effective than other irrigants that may be used in preventing blockages and ledges formation in the root canal. ^29^ Therefore, even without pre-flaring, the Raypex 6 may still provide accurate measurements.

The results showed that the readings when using Raypex 6 (6 ^th^ generation electronic apex locator) are significantly closer to the actual working length than Root ZX (3 ^rd^ generation electronic apex locator) for the with/without coronal pre-flaring groups. This coincides with the results of Pegum Unsal Peker *et al.* who concluded that Raypex 6 is not influenced by the presence of irrigation solutions due to its multi-frequency technology that displays precise results for the WL. ^9^^,^^30^ The 6 ^th^ generation apex locator is proved to be less sensitive to the influence of external factors that increase measuring reliability, ^31^ like the number and taper of the file used in coronal pre-flaring that may influence the enlargement of the coronal portion of the canal. ^32^ The sixth-generation EALs are proven to have a preliminary determination of the canal moistness and based on the constant determined moistness, the sixth-generation EALs adapt the measuring method for either a dry or a wet root canal environment. ^33^

It is important to keep in mind that the accuracy of an electronic apex locator can vary depending on the type of irrigant used, so it is important to use the most appropriate irrigant for the situation at hand. The results of the current study showed the best results with the Raypex 6 apex locator were in dry medium in all conditions of pre-flaring (without pre-flaring & with pre-faring) (T=3.8, P=0.001) and (T=5.4, P=0.001) respectively. This may be due to the measuring changes in canal resistance, as it is easier to obtain accurate measurements in dry root canals. ^34^ Moreover, our findings come in accordance with a study conducted by Koçak *et al*. for working length measurement in dry condition that showed more accurate readings than wet canals. ^35^ However, our findings contradict a previous study conducted by Nayif MM *et al*., (2011) who stated that when using saline as an irrigant, readings were closer to the actual length, whereas those conducted in dry root canals were shorter than the actual working length. ^36^

In accordance with our study, Root ZX apex locator achieved significant results with the CHX in all conditions of pre-flaring (without pre-flaring & with pre-faring): (T=3.85, P=0.001) and (T=3.99, P=0.001) respectively. This may be due to the different electrical conductivities of the irrigants which is defined as the intrinsic ability of the irrigant to conduct the electric current. ^11^ Moreover, single-rooted teeth with single canal orifice were used which may contribute to the lack of difference between the groups with and without coronal pre-flaring. On the contrary, multi-rooted teeth with more canal orifices have a high potential for more anatomical variations and may have differences when coronal pre-flaring was done prior to WL determination. ^16^

Moreover, electroconductivity was enhanced in the current study by using alginate as an embedding material for electronic working length determination. Using the alginate model gives reliable and reproducible results as it has favorable characteristics that mimic the clinical situation by ensuring the required electric circuit for proper measurement of the electronic apex locator. That’s because it mimics the electric resistance of the human periodontal ligament. ^37^ Despite the alginate’s firm consistency, it can remain as a gel that may allow the ions to circulate and promote adequate electroconductivity. Hence it is recommended to use alginate as embedding material for laboratory choices. ^37^^,^^38^ Lucena-Martin *et al.* reported that electronic WL measurements should be concluded within 2 hours after mixing the alginate to minimize the chance of moisture loss. ^39^ While Lipski *et al.* reported that the most accurate readings were obtained within only 30 minutes after mixing the alginate to enhance the electrical conductivity of the used irrigants and EAL. ^40^ Consequently, the technique of alginate usage for only the first 30 minutes of mixing was used in the current study to ensure accuracy.

## Conclusions

In conclusion, the study results suggested that adhering to the endodontic principles in conventional access opening, coronal pre-flaring, and patency are the cornerstone in achieving the most accurate and reproducible working length measurements in Root ZX and Raypex 6 EALs. It is concluded that the irrigant type selection has a major role in the accuracy of the EAL readings. Generally, using the 6 ^th^ generation EAL (Raypex 6) is the most accurate choice for measuring the WL, but when used in a dry medium, it will achieve the most accurate WL measurements. Regarding the 3 ^rd^ generation EAL (Root ZX), it is better to be used with 2% CHX to achieve the most accurate WL of the root canal. Hence, it is very important to know the specific irrigating medium used with each specific EAL to achieve the most accurate WL results.

## Data Availability

figshare: The Effect of Coronal Pre-flaring and Root Canal Irrigant on Apex Locators Accuracy: In-Vitro Study, https://doi.org/10.6084/m9.figshare.22492354.v4. ^41^ This project contains the following underlying data:
- Apex locators results.xlsx - Microscope images for cases 1-6 Due to the size of the original microscopy images, they are not all able to be uploaded to a public repository. Readers and reviewers can request access to further images from the corresponding author ( srhussein@iau.edu.sa). figshare: The Effect of Coronal Pre-flaring and Root Canal Irrigant on Apex Locators Accuracy: In-Vitro Study, https://doi.org/10.6084/m9.figshare.22492354.v4. ^41^ This project contains the following extended data:
- Manuscript tables & figures - Additional images Data are available under the terms of the Creative Commons Attribution 4.0 International license (CC-BY 4.0).
