# The Effect of Coronal Pre-flaring and Type of Root Canal Irrigation on Working Length Accuracy Using Electronic Apex Locators

**DOI:** 10.12688/f1000research.133288.2

**Published:** 2023-09-04

**Authors:** Shimaa Rifaat, Abdullah Aljami, Turki Alshehri, Shahad T. Alameer, Alhanoof Aldossary, Wejdan Almutairi, Mulham N. Almaliki, Faraz A. Farooqi, Noha Taymour

**Affiliations:** 1 Department of Restorative Dental Sciences, College of Dentistry, Imam Abdulrahman Bin Faisal University, P.O Box 1982, Dammam, 31411, Saudi Arabia; 2 College of Dentistry, Imam Abdulrahman Bin Faisal University, P.O. Box 1982, Dammam, 31411, Saudi Arabia; 3 Department of Dental Education, College of Dentistry, Imam Abdulrahman Bin Faisal University, P.O. Box 1982, Dammam, 31411, Saudi Arabia; 4 Department of Substitutive Dental Sciences, College of Dentistry, Imam Abdulrahman Bin Faisal University, P.O. Box 1982, Dammam, 31411, Saudi Arabia

**Keywords:** Working length, Root ZX apex locator, Raypex 6 apex locator, irrigating solution, coronal pre-flaring, sodium hypochlorite, chlorhexidine, dry medium.

## Abstract

**Background:** Successful root canal treatment is influenced by the apical extent of root canal preparation and the eventual root canal filling. Achieving the full working length until the apical constriction, which is usually 0.5 – 1 mm shorter than the anatomical apex, is crucial. Electronic apex locators were used to detect the working length more accurately. There are six generations of electronic apex locators in the market. The selection of the appropriate irrigation with each apex locator for accurate working length determination is not fully investigated.

**Methods:** The actual working lengths of 120 freshly extracted human single-rooted teeth were measured and compared with their working lengths using 3 ^rd^ generation (Root ZX) followed by 6 ^th^ generation (Raypex 6) apex locators in dry medium, presence of 5.25% sodium hypochlorite, and 2% chlorhexidine, without coronal pre-flaring and after coronal pre-flaring using the same irrigating media. Data were collected, tabulated, and afterward analyzed using one-way ANOVA with post-hoc to evaluate the significant difference in average working length between actual working length, Root ZX, and Raypex 6 apex locator working lengths accuracy.

**Results:** The significant results were shown in roots that were coronally pre-flared and their working lengths were measured in a dry medium using Raypex 6 apex locator. While using the Root ZX apex locator, the most accurate results were shown in roots that were coronally pre-flared and their working lengths were measured while using a chlorhexidine irrigating solution.

**Conclusions:** It is concluded that it is very important to know the specific irrigating medium to be used with each specific electronic apex locator to achieve the most accurate working length results.

## Introduction

Successful root canal treatment (RCT) is influenced by the apical extent of root canal preparation and eventual root canal filling. ^1^ The suggested endpoint for instrumentation and obturation is apical constriction. ^2^ Apical constriction is defined as the minor root canal diameter. Histologically, it represents the transitional point between the pulpal and periodontal tissues at the cemento-dentinal junction (CDJ). According to anatomical research, the apical constriction is 0.5–1.0 mm from the exterior or the main foramen (anatomical apex). ^3^ Therefore, full WL provides a clean barrier, protecting the periodontium from bacterial invasion. ^3^ Moreover, underfilling or overfilling of the canals is one of the reasons for endodontic treatment failures. ^4^ ^–^ ^6^

Accordingly, the determination of WL is a critical step in endodontic treatment. ^3^ There are many ways to determine WL: periapical X-rays (PAs), bleeding points, electronic apex locators (EALs), and the tactile sensation of the operator. ^7^ The most commonly used method is to combine EALs and periapical radiographs. ^8^ Thus, a reliable apex locator should be used to reduce patient exposure to X-rays. Six generations of EALs are used to detect WL. The most popular system is the third-generation apex locator. ^9^ It uses two frequencies and measures the difference between them; however, the type of moisture present may affect reading accuracy. ^9^ ^,^ ^10^ On the other hand, the 4 ^th^ generation uses five frequencies, but they make mathematical measurements rather than measurements according to a database. ^9^ ^,^ ^10^ However, there are no marked differences in reading accuracy between older apex locator generations. The 5 ^th^ generation uses a database of canal electrical features and compares them using a mathematical process. New technological advancements have led to the sixth generation of EAL, in which a steady algorithm was created according to the canal’s moisture properties. Furthermore, working length measurements are more reproducible and accurate than those of previous generations. ^9^

The use of irrigating media to determine WL has been reported in the literature. Keeping the root canal dry or moist with an irrigator while using apex locators has been questioned by many dental practitioners as to whether it increases the accuracy rate of EAL. Using 5.25% sodium hypochlorite (NaOCl) during RCT meets the prime principles of endodontics, chemomechanical cleaning, and root canal shaping. NaOCl is also used as an antibacterial agent to dissolve organic components of the root canal system. ^11^

Chlorhexidine gluconate (CHX; 2%) is a broad-spectrum antiseptic cationic agent that exists in two forms: gel (CHX-G) and solution (CHX-S). However, it cannot dissolve organic materials as 5.25% NaOCl does. ^11^ A 2% CHX solution has been used in endodontics as an irrigating material or intracanal medicament because it has a broad spectrum of antimicrobial activity and a lower cytotoxic effect than NaOCl. While showcasing its effective clinical performance, lubricating characteristics, and gel-like rheological action, it additionally inhibits metalloproteinase, maintains chemical stability, avoids clothing staining, lacks odor, dissolves in water, and, thanks to its cationic structure, possesses a distinct attribute referred to as substantivity (remaining antimicrobial activity). ^12^ ^,^ ^13^ It is well established that the chemomechanical procedure can be enhanced if followed by the use of an antibacterial intracanal medication such as chlorhexidine (CHX), especially in cases of exudation, hemorrhage, perforation, root resorption, trauma, or insufficient root development. ^13^

Coronal pre-flaring of root canals has several advantages that can be reflected in the cleaning and shaping processes, including making it easier to place manual and rotary equipment in the apical region of the root canals. ^14^ ^,^ ^15^ The results indicated that the pre-flaring procedure provided more accurate measurements in most cases. ^16^

Few studies have been conducted to evaluate the effect of irrigating solutions on the WL reading accuracy of various EALs in the presence or absence of coronal pre-flaring. Hence, the present study aimed to compare WL accuracy using 3 ^rd^ generation (Root ZX) and 6 ^th^ generation (Raypex 6) EALs in single-rooted teeth in the presence of different irrigation media: dry medium, 5.25% sodium hypochlorite (NaOCl), and 2% chlorhexidine (CHX) in root canals with or without coronal pre-flaring.

### Hypothesis

No significant difference in the accuracy of WL determination was detected by coronal pre-flaring or the type of irrigant, which varied with the different generations of EALs.

## Methods

### Ethical approval

The study complied with the Declaration of Helsinki, and the research protocol was approved by the Restorative Dental Science Department, Imam Abdulrahman Bin Faisal University, College of Dentistry. Ethical approval was obtained from the Institutional Review Board of Imam Abdulrahman Bin Faisal University (IRB 2022-02-171) on April 12, 2022.

Teeth were collected during disposal after receiving signed consent from dental surgery or oral procedure patients, who authorized the hospital to use their discretion in their disposal and to be used for research purposes if needed.

### Sample size calculation

A power analysis was performed using the clinical sample size for this study. Means and standard deviations were obtained from previously published literature, ^11^ which were (0.64 ± 0.54) and (0.33 ± 0.22). The power of the sample was set at 90%, and the significance level was set at 0.05. Hence, the calculated total sample size was 109, which was increased to 120 for more precise results.

### Sample selection

The study began on April 17, 2022. In total, 120 freshly extracted human single-rooted teeth were used in this study. They were collected anonymously without exposing the patients’ data, and were used only for this in vitro study. The samples were collected and immersed in 5.25% sodium hypochlorite (NaOCl) for 2 hours for disinfection. ^3^ Afterwards, the samples were stored in normal saline until further use. Root surfaces and apical regions were examined under a dental operating microscope (OMS 1950 Dental Microscope, USA) at 25× magnification. Teeth of comparable lengths and completely formed apices were included in the current study. Teeth with any possible fractures or apex immaturity were excluded from the study. The teeth were radiographed in the mesiodistal and buccolingual directions to exclude the absence of root resorption or canal curvature. Only root canals with a curvature of 0–5° were included in the study. Teeth with calcified canals, more than one canal, apical blockage, internal or external resorption, or caries were excluded from this study.

### Sample preparation

The teeth were examined using periapical X-rays with a 0.08-second exposure time. Scaling of the teeth was performed using an ultrasonic scaler (Dentsply Sirona, ProUltra Piezo Ultrasonic Handpiece) and then stored in 0.9% normal saline until use. The teeth were flattened with a diamond disc of 1 mm thickness (Hi-Tech diamond disc bur) to obtain a reliable reference point based on the findings of Jakobson et al. that the rubber stopper on the file should be placed on a flat surface to limit the possibility of errors in research with EALs, which ensured that the study findings were not influenced. ^17^ WL was evaluated by two evaluators, who were instructed to use the same criteria to evaluate and assess the parameters of the current study. Cohen’s Kappa test was applied to ensure agreement and consistency between the two evaluators’ WL evaluations. A value of 0.82 was interpreted as a high level of agreement or reliability between the two evaluators.

### Working length measurements without coronal pre-flaring

Conventional access opening without coronal pre-flaring was performed. Apical patency was assessed using K-file #10 (Dentsply M-access K-file). The actual WL was measured in millimeters by two calibrated evaluators using an endodontic microscope (OMS 1950 Dental Microscope) at 25× magnification. Any tooth with an initial file larger than the #15 K-file (Dentsply M-Access K-File) was excluded. The file was placed beyond the apical constriction and retrieved until it was flushed through the apical foramen. 0.5 mm was subtracted from the total, and the final measurement was considered as the actual WL. ^2^ ^,^ ^8^ A double rubber stopper was used. ^3^ Using freshly mixed alginate, each tooth was mounted at the cemento-enamel junction (CEJ) level using freshly mixed alginate.

### Working length determination

The WL in millimeters for each tooth was measured using the 3 ^rd^ generation EAL Root ZX (J. Morita Corp., Kyoto, Japan), followed by the 6 ^th^ generation EAL Raypex 6 (VDW, Munich, Germany). Apex locators were used while the teeth were dry, using 5.25% NaOCl and 2% CHX. Between each irrigating solution and the next, distilled water was used to neutralize each irrigating effect before using the next one. The canals were dried using paper points. All measurements were taken by two calibrated examiners. A K-file size #15 (Dentsply M-access K-File) was inserted inside the canal, a lip clip was placed inside a fresh alginate mix, and a file holder was placed on the file.

The file was fixed to the apex locators: third generation EAL Root ZX (J. Morita Corp., Kyoto, Japan) and sixth generation EAL Raypex 6 (VDW, Munich, Germany) for 5 seconds before recording measurements. The measurements were recorded when the file reached the mid-green area on the EALs’ screen. ^18^

### Working length measurements with coronal pre-flaring

**Figure 1.**
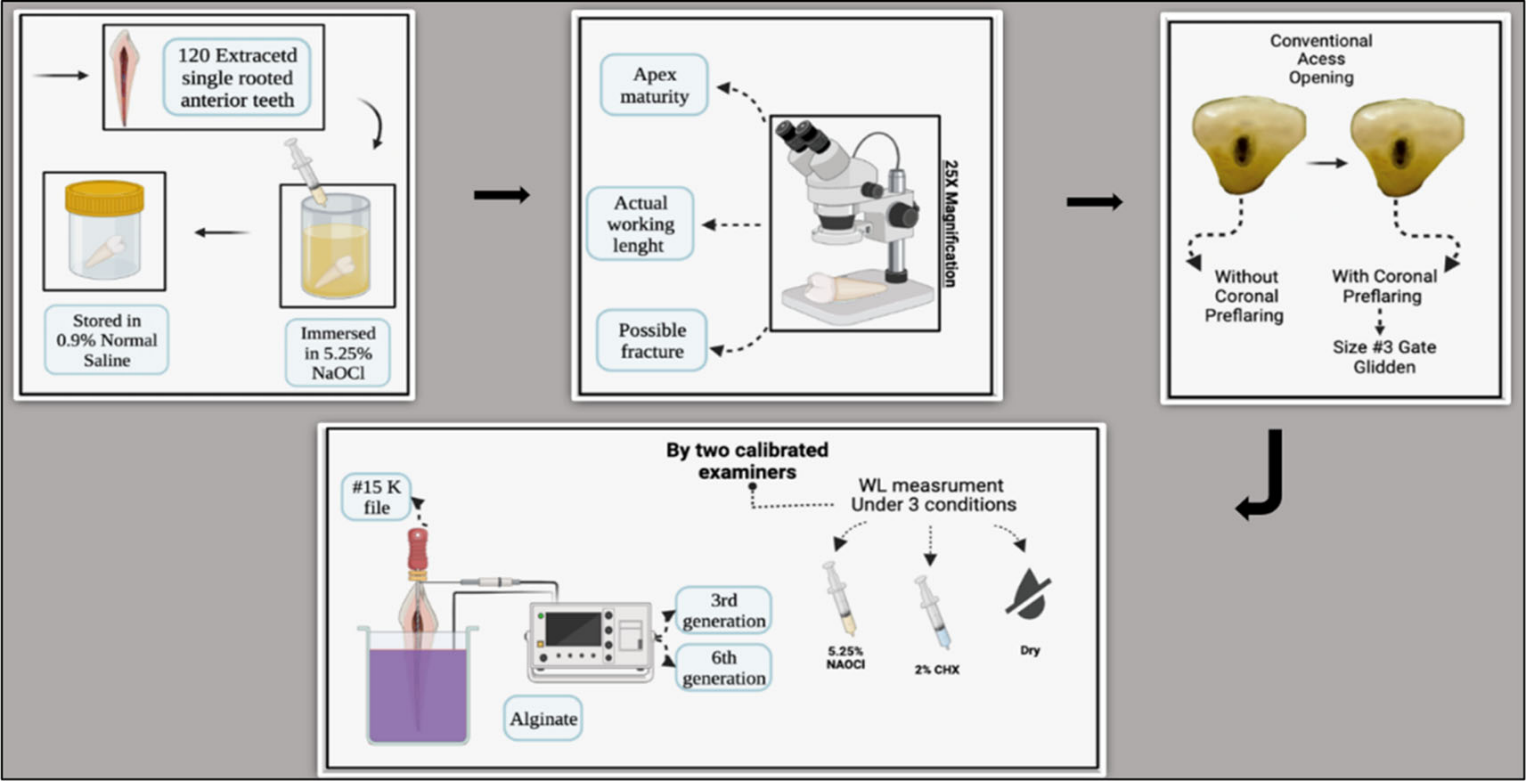
Schematic drawing showing the following steps for the study: tooth selection, tooth storage, measuring the actual working length under the microscope, access opening preparation without coronal pre-flaring followed by access opening preparation with coronal pre-flaring, teeth mounted in alginate, holding the file in the double stopper technique, and measuring the working length (WL) in millimeters using different irrigation media (dry medium, 5.25% NaOCl, 2% CHX) with 3 ^rd^ and 6 ^th^ generation EALs.

All teeth were collected, and coronal pre-flaring of the root canals was performed. An access opening was prepared for each tooth. The penetration depth of the gate-glidden drills was as follows: #3 to the canal orifice, #2 to the coronal third, and maximum to the coronal half of the canal to avoid perforations and achieve straight-line access. ^19^ Measuring the WL in millimeters of all teeth was repeated after coronal pre-flaring with both Root ZX (3 ^rd^ generation) and Raypex 6 (6 ^th^ generation) apex locators using dry medium, 5.25% NaOCl, and 2% CHX irrigating solutions, as shown in Figure 1.

### Statistical analysis

The data were initially recorded in an Excel spreadsheet and then transferred to SPSS version 24 (IBM, Inc., Chicago, IL, USA). The means and standard deviations were calculated and presented in tables as descriptive statistics. Comparisons between the irrigant solutions for Root ZX and Raypex 6 EAL were performed using an independent sample t-test and ANOVA. Where ANOVA was significant, multiple comparisons were made using Tukey’s post hoc test. A comparison of WL between the 3 ^rd^ (Root ZX) and 6 ^th^ generation (Raypex 6) apex locators in teeth without coronal pre-flaring and with coronal pre-flaring in all media was also performed using an independent sample t-test. Statistical significance was set at p < 0.05.

## Results

**Figure 2.**
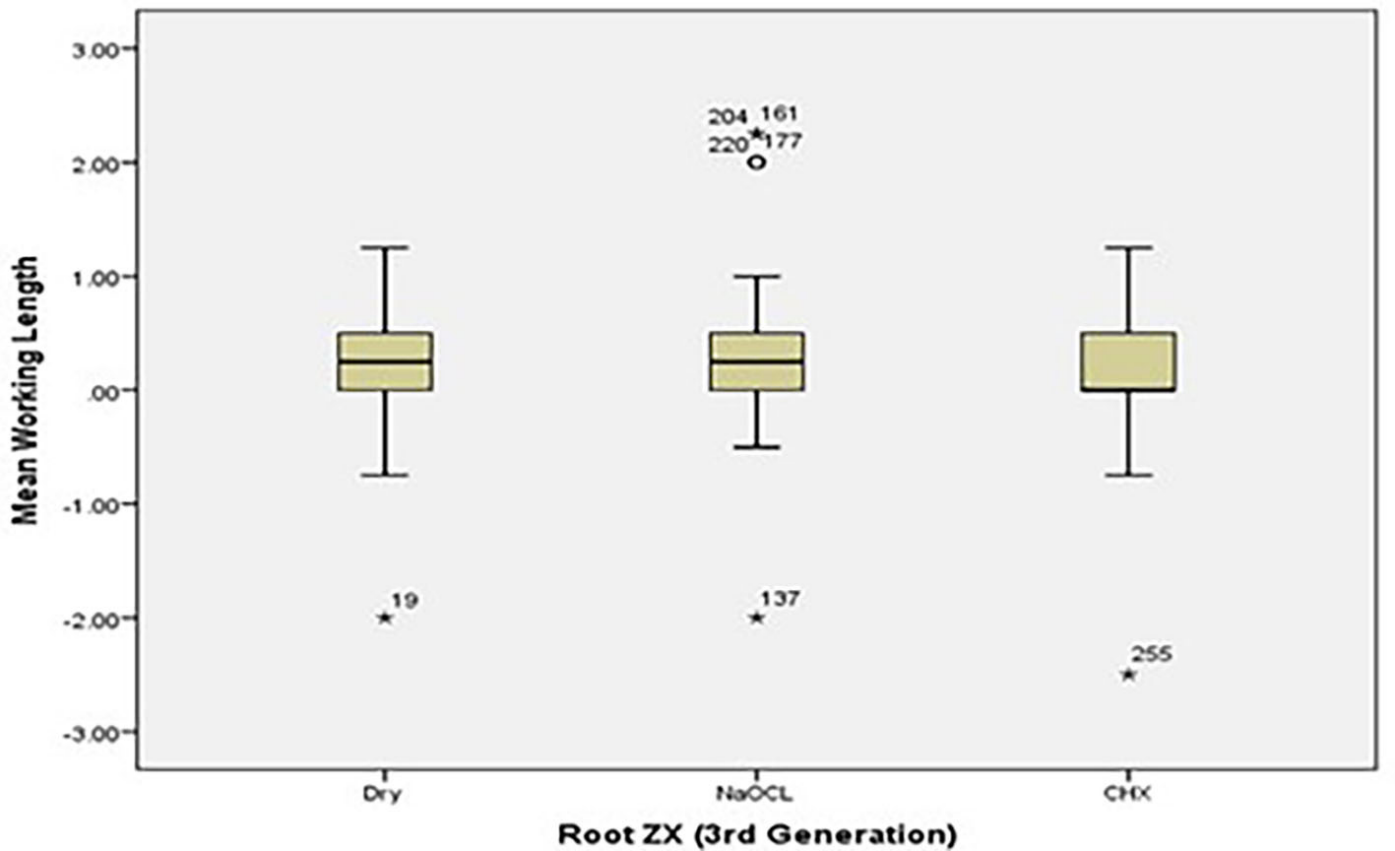
Working length measurements for Root ZX for all irrigating solutions without canal pre-flaring.

**Figure 3.**
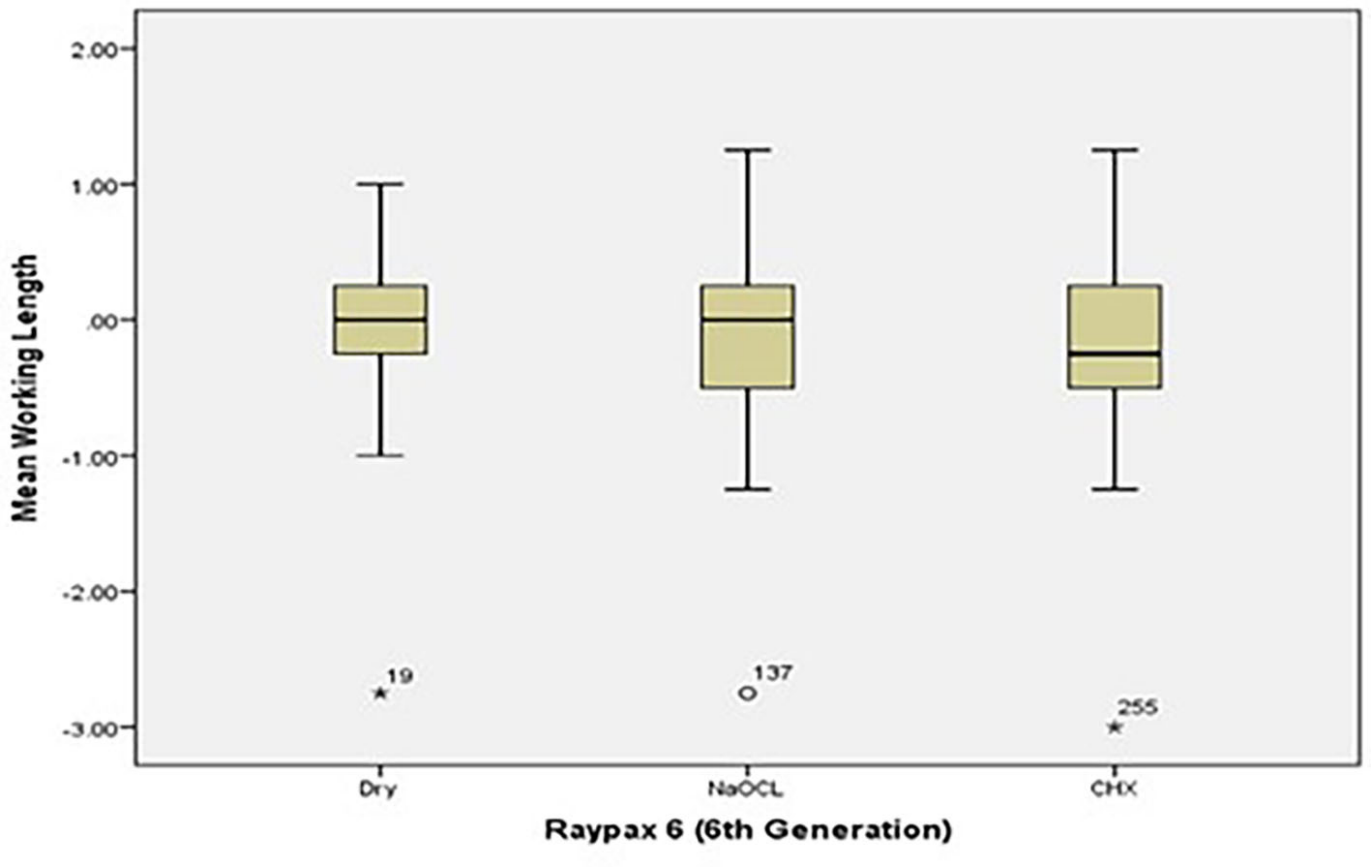
Working length measurements for Raypex 6 for all irrigating solutions without coronal pre-flaring.

Table 1 compares the mean WL of Root ZX and Raypex 6 EALs between the irrigating solutions and within the irrigating solutions without coronal pre-flaring. Mean length measurements for Root ZX in CHX were significantly closer to the actual WL (0.087 ± 0.445) than the other two irrigants; the least close measurement from the WL was with NaOCl (0.252 ± 0.553), and the difference was statistically significant (p = 0.025). However, with Raypex 6, the dry medium should have the closest readings to WL, and the overall mean difference among the irrigants used was insignificant. Within each irrigating solution, the mean length of Raypex 6 was close to the actual WL and statistically significant. Moreover, the dry medium showed the most accurate WL with the Raypex 6, the NaOCl demonstrated the most accurate WL with the Raypex 6, and the CHX displayed the most accurate WL with Root ZX in all non-pre-flaring conditions. Figures 2 and 3 present the pre-flaring WL measurements for Root ZX and Raypex 6 for all irrigating solutions. The median lengths for dry medium and NaOCl were almost the same, whereas the median length for CHX significantly decreased and was closer to the actual WL; asterisks indicate outliers.

**Table 1.** Comparison of mean working length (WL) of Root ZX and Raypex 6 EALs in the presence of Dry medium, 5.25% NaOCl, and 2% CHX media without coronal pre-flaring.

	Without pre-flaring			p-values of ANOVA
	Dry	NaOCl	CHX	
**Root ZX (3** ^**rd**^ **generation)**	0.146 ± 0.399	0.252 ± 0.553 ^a^	0.087 ± 0.445 ^a^	F = 3.7, p = 0.025 *
**Raypex 6 (6** ^**th**^ **generation)**	-0.074 ± 0.486	-0.121 ± 0.506	-0.161 ± 0.534	F = 0.861, p = 0.42
**p-values of t-test**	T = 3.8, p = 0.001 *	T = 5.4, p = 0.001 *	T = 3.85, p = 0.001 *	

* Statistically significant at 0.05.

^a^ Significant differences between solutions horizontally.

Similarly, Table 2 presents the mean WL difference among the solutions for both apex locators in the presence of coronal pre-flaring. The mean WL for the Root ZX locator differed significantly among the solutions (p = - 0.038). The closest mean to the actual WL was recorded with CHX (0.068 ± 0.586), whereas the mean that differed the most from the actual WL was recorded in a dry medium (0.269 ± 0.621). Likewise, the most accurate mean WL for the Raypax 6 locator was recorded in a dry medium (-0.464 ± 0.641), the least accurate WL was recorded with NaOCl (-0.174 ± 0.584), and the difference between the actual WL and the mean was statistically significant. Both apex locators differed significantly between irrigation solution groups.

**Table 2.** Comparison of mean WL of Root ZX and Raypex 6 EALs in the presence of Dry medium, 5.25 % NaOCl, and 2% CHX media with coronal pre-flaring.

	With pre-flaring			p-values of ANOVA
	Dry	NaOCl	CHX	
**Root ZX (3** ^**rd**^ **generation)**	0.269 ± 0.621 ^a^	0.206 ± 0.64	0.068 ± 0.586 ^a^	F = 3.28, p = 0.038 *
**Raypex 6 (6** ^**th**^ **generation)**	-0.174 ± 0.584 ^a^	-0.464 ± 0.641 ^a^	-0.28 ± 0.74	F = 5.87, p = 0.003 *
**p-values**	T = 5.4, p = 0.001 *	T = 8.02, p = 0.001 *	T = 3.99, p = 0.001 *	

* Statistically significant at 0.05.

^a^ Significant differences between solutions horizontally.

**Figure 4.**
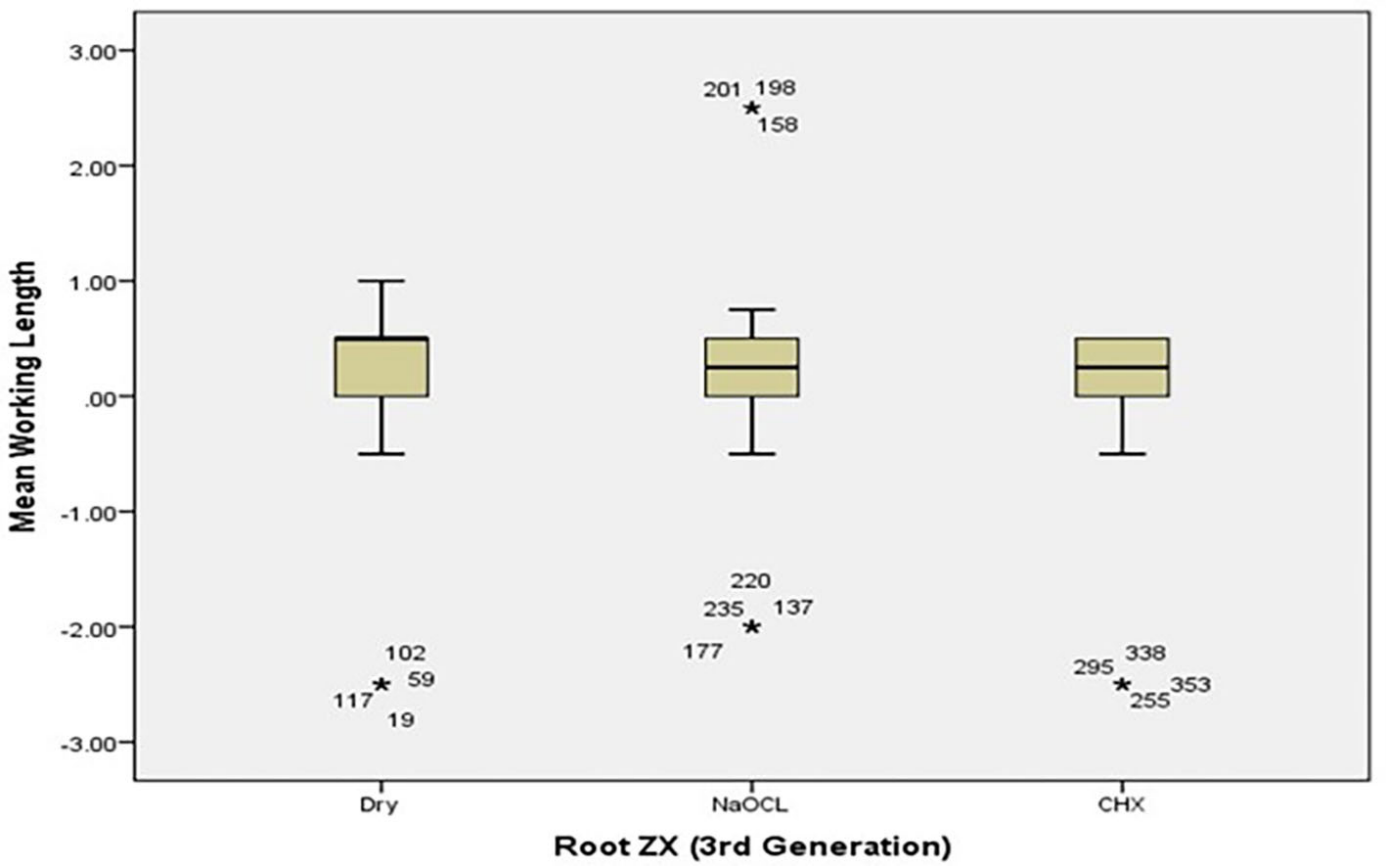
Working length measurements for Root ZX for all irrigating solutions with coronal pre-flaring.

**Figure 5.**
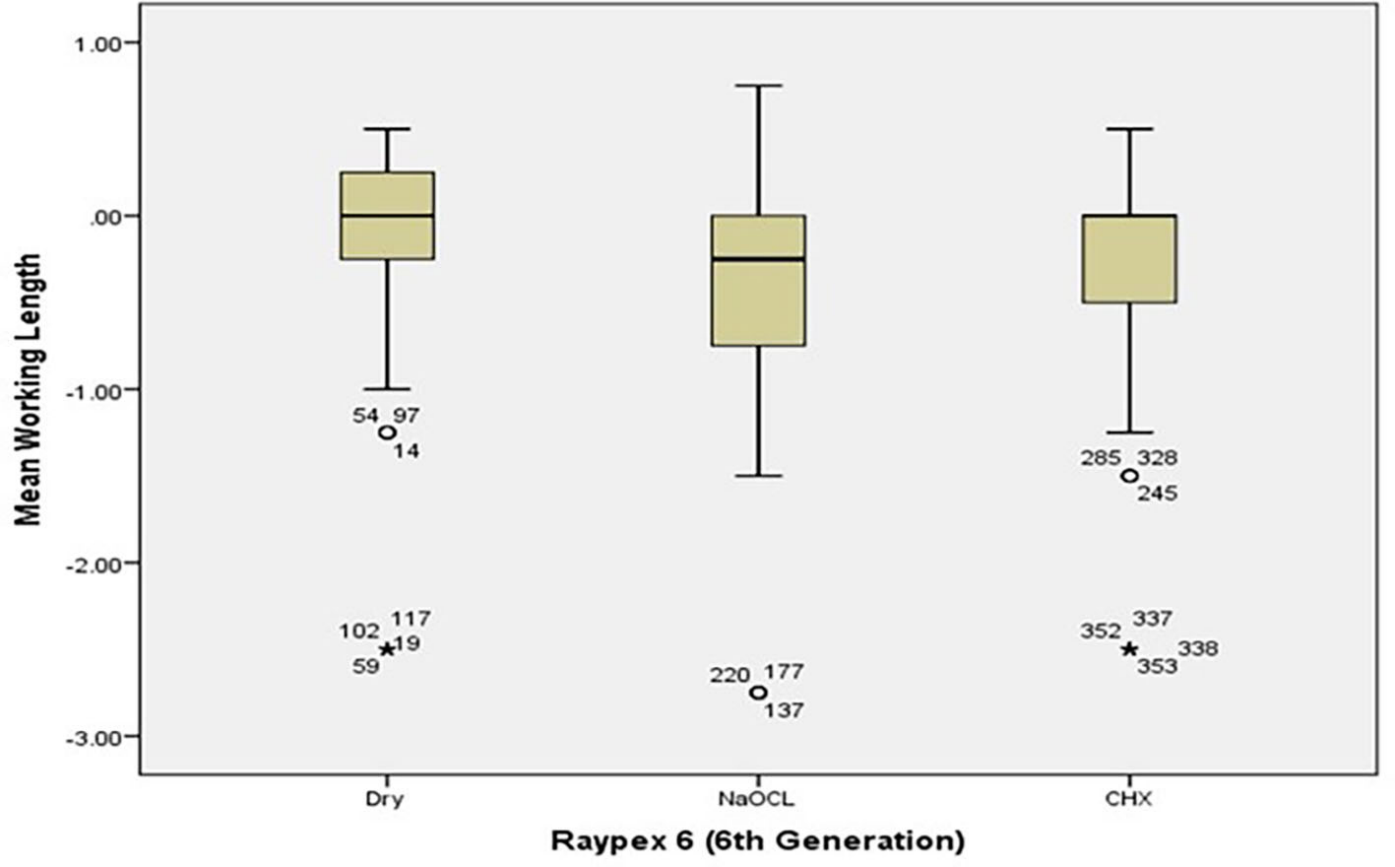
Working length measurements for Raypex 6 for all irrigating solutions with coronal pre-flaring.

Moreover, the dry medium displayed the most accurate WL with Raypex 6, followed by NaOCl achieved the most accurate WL with Root ZX, and CHX showed the most accurate WL with Root ZX in all coronal pre-flaring conditions ( Table 2). The box plots in Figures 4 and 5 show the median WL spread of the measurements. Figure 4 shows that the median WL in NaOCl and CHX media was almost equal, but significantly different in the dry medium. Figure 5 illustrates a similar pattern for the Raypax 6 apex locator, where the asterisks in each box plot refer to outliers.

The WL for both apex locators was then compared between the presence and absence of coronal pre-flaring, as presented in Table 3. In both cases (with and without coronal pre-flaring), the mean length did not differ significantly for either apex locator in dry medium (p = -0.072), but the closest mean WL to the actual WL was recorded in irrigation groups without coronal pre-flaring (0.146 ± 0.39, -0.074 ± 0.48, respectively). Similarly, in the NaOCl solution, the most accurate mean WL was recorded without coronal pre-flaring media for the Raypex 6 apex locator (0.121 ± 0.50), and the difference was statistically significant (p = -0.001). The closest mean WL to the actual WL in CHX for Root ZX was found in pre-flaring media (0.068 ± 0.58), whereas for Raypax 6, it was the closest in the same media (0.28 ± 0.74), but the difference was not statistically significant. When compared regardless of the irrigation solution, the most accurate mean WL to the actual WL was recorded in the absence of coronal pre-flaring media groups for both apex locators (0.161 ± 0.37, -0.118 ± 0.44, respectively), and the difference was only statistically significant for the Raypex 6 apex locator (p = -0.005).

**Table 3.** Comparison of WL of roots irrigated with different irrigating media under all flaring conditions (with or without coronal pre-flaring).

Groups	Dry		NaOCl		CHX		Overall	
	Root ZX	Raypex 6	Root ZX	Raypex 6	Root ZX	Raypex 6	Root ZX	Raypex 6
**Without pre-flaring**	0.146 ± 0.39	-0.074 ± 0.48	0.252 ± 0.55	-0.121 ± 0.50	0.087 ± 0.44	-0.161 ± 0.53	0.161 ± 0.37	-0.118 ± 0.44
**With pre-flaring**	0.269 ± 0.62	-0.174 ± 0.58	0.206 ± 0.64	-0.464 ± 0.64	0.068 ± 0.58	-0.28 ± 0.74	0.181 ± 0.54	-0.30 ± 0.57
**p-value**	T= -1.807, p = 0.072	T= -1.407, p = 0.156	T = 0.598, p = 0.550	T = 4.5, p = 0.0001 *	T = 0.281, p = 0.779	T = 1.41, p = 0.159	T = 3.14, p = 0.734	T = 2.86, p = 0.005 *

* Statistically significant at 0.05.

## Discussion

The establishment of an accurate WL is a crucial step during root canal treatment, particularly in cases of anatomical limitations. It is useful when used with radiographs to ensure proper determination of the canal WL. ^20^ The coronal pre-flaring of root canals provides many advantages during meticulous cleaning and shaping procedures, such as facilitating the insertion of manual and/or rotary files into the apical third of the root canals by removing cervical dentin interferences. ^21^ In addition, coronal flaring improved the flow of the irrigating solution within the root canal, minimizing the risk of bacterial invasion into the periapical tissue and reducing the risk of canal debris and irrigant extrusion during the root canal preparation procedure. ^22^ ^,^ ^23^

In recent years, a growing body of evidence has suggested a correlation between the type of root canal irrigating solution used and the success of coronal pre-flaring efficacy. Moreover, the use of root canal irrigants is a major contributor to the success of endodontic treatment. Some studies have shown a correlation between the irrigant and the root canal sealer used to ensure a proper hermetic seal, ^24^ while others have discussed the effect of the proper irrigant on the accuracy of WL determination. ^25^

The current investigation evaluated the influence of coronal pre-flaring on the accuracy of actual working length determination, and the results showed that the canals without coronal preflaring at the coronal end provided more consistent and accurate outcomes than canals that were pre-flared ( Table 3). These results are consistent with those of a previous study conducted by João Marcelo da Silva Teixeira et al. (2012), who concluded that the use of Gates Glidden burs for cervical pre-flaring did not significantly influence the accuracy of apical placement of the apex locator when determining the actual WL because of insufficient removal of coronal dentin when compared with the rotary system for preparing coronal pre-flaring. ^26^ However, this contradicts a previous study that showed that pre-flaring improved the efficiency of EALs in mandibular and anterior root canals. ^16^

When NaOCl was used as an irrigant, significant results were obtained with Raypex 6, even without coronal pre-flaring. This coincided with the results of Elnaghy et al. (2017), who found that the use of 5.25% sodium hypochlorite (NaOCl) as an irrigant without coronal pre-flaring was associated with greater success rates than a 2% chlorhexidine (CHX) solution. This may be due to the good electrical conductivity of NaOCl, which contributes to the accurate detection of Raypex 6 at the apical constriction. Moreover, this may be due to the advanced technology of Raypex 6, which can accurately operate under different canal conditions, including the presence of debris and/or obstructions in the canal. ^9^ Furthermore, researchers also noted that sodium hypochlorite irrigants were more effective than other irrigants that may be used to prevent blockage and ledge formation in the root canal. ^27^ Therefore, even without pre-flaring, Raypex 6 may provide accurate measurements.

The results showed that the readings when using Raypex 6 (6 ^th^ generation EAL) were significantly closer to the actual WL than when using Root ZX (3 ^rd^ generation EAL) for the groups with and without coronal pre-flaring. This coincides with the results of Pegum Unsal Peker et al., who concluded that Raypex 6 is not influenced by irrigation solutions because of its multifrequency technology, which shows precise WL results. ^9^ ^,^ ^28^ The 6 ^th^ generation apex locator is believed to be less sensitive to external factors that increase measuring reliability, ^29^ such as the number and taper of the files used in coronal pre-flaring, which may influence the enlargement of the coronal portion of the canal. ^30^ The sixth generation EALs have been proven to have a preliminary determination of canal moistness, and based on the constant determined moisture, the sixth-generation EALs adapt the measuring method for either a dry or wet root canal environment. ^31^

It is important to remember that the accuracy of an EAL can vary depending on the type of irrigant used. Hence, it is important to use the most appropriate irrigant for the situation at hand. The current study showed the best results with the Raypex 6 apex locator in the dry medium under all conditions of pre-flaring (without pre-flaring and with pre-flaring) (T = 3.8, p = 0.001) and (T = 5.4, p = 0.001), respectively. This may be due to the ability to detect changes in canal resistance, as it is easier to obtain accurate measurements in dry root canals. ^32^ Moreover, our findings agree with those of a study conducted by Koçak et al. for WL measurements in dry conditions, which showed more accurate readings than wet canals. ^33^ However, our findings contradict those of a previous study by Nayif et al. (2011), who stated that when saline was used as an irrigant, readings were closer to the actual length, whereas those conducted in dry root canals were shorter than the actual WL. ^34^

In accordance with our study, the Root ZX apex locator achieved significant results with CHX in all conditions of pre-flaring (without pre-flaring and with pre-flaring) (T = 3.85, p = 0.001) and (T = 3.99, p = 0.001), respectively. This may be because of the different electrical conductivities of the irrigants, which are defined as the intrinsic ability of the irrigant to conduct electric currents. ^11^ Moreover, single-rooted teeth with a single canal orifice were used, which may have contributed to the lack of difference between the groups with and without coronal pre-flaring. In contrast, multirooted teeth with more canal orifices have a higher potential for more anatomical variations and may have differences when coronal pre-flaring is performed prior to WL determination. ^16^

Moreover, electro-conductivity was enhanced in the current study by using alginate as an embedding material to determine electronic WL. The alginate model provides reliable and reproducible results as it has favorable characteristics that mimic the clinical situation by ensuring the required electric circuit for proper measurement of the EAL. This is because they mimic the electrical resistance of the human periodontal ligament. ^35^ Despite the consistency of alginate, it can remain a gel that allows ions to circulate and promotes adequate electro-conductivity. Hence, it is recommended that alginate be used as an embedding material in laboratory applications. ^35^ ^,^ ^36^

Lucena-Martin et al. reported that electronic WL measurements should be concluded within 2 hours after mixing the alginate to minimize moisture loss. ^37^ Lipski et al. reported that the most accurate readings were obtained within 30 minutes after mixing alginate to enhance the electrical conductivity of the irrigants and EAL. ^38^ Consequently, alginate was used for only the first 30 minutes of mixing in the current study to ensure accuracy.

The null hypothesis was partially rejected because of the differences in the results obtained from the different irrigation solutions used with each EAL. However, there was no significant difference between groups with and without coronal flaring. This was within the limitations of the current study on using single-root teeth with an initial file size of 15, while using one type of irrigant at a time to be used as a benchmark for future studies. The study’s limitations included using curved canal teeth with various degrees of curvature and larger apical foramen sizes, which may have affected the results. Furthermore, the results of using one irrigant at a time may differ.

Moreover, agitation of the irrigated solution may affect the electrical conductivity and, hence, the reading accuracy of the EAL. Irrigation volume, concentration, temperature, and application method may also influence reading accuracy. In addition, applying the current study in vivo in the presenc of patient’s body fluids may affect the results.

## Conclusions

In conclusion, the study results suggest that adherence to the endodontic principles of conventional access opening, coronal pre-flaring, and patency are the cornerstones for achieving the most accurate and reproducible WL measurements in Root ZX and Raypex 6 EALs. It was concluded that irrigant-type selection plays a major role in the accuracy of EAL readings. Generally, using the 6 ^th^ generation EAL (Raypex 6) is the most accurate choice for measuring WL. However, it provides the most accurate WL measurements when used in a dry medium. Regarding the 3 ^rd^ generation EAL (Root ZX), it is better to use it with 2% CHX to achieve the most accurate WL of the root canal. Hence, it is important to know the specific irrigating medium used with each specific EAL to achieve the most accurate WL results.

## Data availability

### Underlying data

Figshare: The Effect of Coronal Pre-flaring and Root Canal Irrigant on Apex Locator Accuracy: An in vitro Study ( https://doi.org/10.6084/m9.figshare.22492354.v4. ^39^)

This project contained the following underlying data:

• Apex locators results.xlsx
• Microscope images for cases 1–6

Due to the size of the original microscopy images, they were not uploaded to a public repository. Readers and reviewers can request additional images from the corresponding author ( srhussein@iau.edu.sa).

### Extended data

Figshare: The Effect of Coronal Pre-flaring and Root Canal Irrigant on Apex Locator Accuracy: An in vitro Study ( https://doi.org/10.6084/m9.figshare.22492354.v4. ^39^)

This project contains the following extended data:

• Manuscript tables and figures
• Additional images

Data are available under the terms of the Creative Commons Attribution 4.0, International License (CC-BY 4.0).
