# Supplementary material for: The Effect of Coronal Pre-flaring and Type of Root Canal Irrigation on Working Length Accuracy Using Electronic Apex Locators
Source: F1000Res. 2023 Oct 24;12:533. Originally published 2023 May 23. [Version 3] doi: 10.12688/f1000research.133288.3 (PMC10643884; doi:10.12688/f1000research.133288.3)
Supplement: Peer review history

# Reviewer report: Reviewer response for version 3

**DOI:** 10.5256/f1000research.157772.r217941

**Published:** 2023-11-13

**Authors:** Amira Salem

**Affiliations:** 1 Sir John Walsh Research Institute, Faculty of Dentistry, University of Otago, Dunedin, Otago, New Zealand; 2 Blis Technologies Limited, Dunedin, New Zealand

The authors have addressed all of the reviewer's concerns and clarified it in the discussion and recommendation.

Is the work clearly and accurately presented and does it cite the current literature?

Yes

If applicable, is the statistical analysis and its interpretation appropriate?

Partly

Are all the source data underlying the results available to ensure full reproducibility?

No source data required

Is the study design appropriate and is the work technically sound?

Yes

Are the conclusions drawn adequately supported by the results?

Partly

Are sufficient details of methods and analysis provided to allow replication by others?

Partly

Reviewer Expertise:

Endodontics, microbiology, molecular biology, biotechnology, bioengineering, product development, drug delivery

I confirm that I have read this submission and believe that I have an appropriate level of expertise to confirm that it is of an acceptable scientific standard.

# Reviewer report: Reviewer response for version 3

**DOI:** 10.5256/f1000research.157772.r217942

**Published:** 2023-10-27

**Authors:** Rania El Backly

**Affiliations:** 1 Endodontics, Conservative Dentistry Department and Tissue Engineering Laboratories, Faculty of Dentistry, Alexandria University, Alexandria, Alexandria Governorate, Egypt

I believe the authors have made substantial revisions and I can endorse the manuscript for indexing.

Is the work clearly and accurately presented and does it cite the current literature?

Partly

If applicable, is the statistical analysis and its interpretation appropriate?

Partly

Are all the source data underlying the results available to ensure full reproducibility?

No source data required

Is the study design appropriate and is the work technically sound?

Yes

Are the conclusions drawn adequately supported by the results?

Yes

Are sufficient details of methods and analysis provided to allow replication by others?

Partly

Reviewer Expertise:

NA

I confirm that I have read this submission and believe that I have an appropriate level of expertise to confirm that it is of an acceptable scientific standard.

# Reviewer report: Reviewer response for version 2

**DOI:** 10.5256/f1000research.153312.r203786

**Published:** 2023-09-27

**Authors:** Rania El Backly

**Affiliations:** 1 Endodontics, Conservative Dentistry Department and Tissue Engineering Laboratories, Faculty of Dentistry, Alexandria University, Alexandria, Alexandria Governorate, Egypt

In general:

 The authors have attempted to address the previous comments however, some changes still need to be made for the manuscript to be suitable for publication. All the responses to the comments should be clearly highlighted in the main manuscript file AND mentioned in the responses letter especially the page no. and line no. where these corrections were made.

***New comments are bolded.***

 Introduction:

- The rationale of the study is unclear. Coronal pre-flaring is a routine part of endodontic instrumentation and the irrigants used in this study are routinely used in everyday clinical practice in association with apex locators, so why are the authors assuming that these variables will affect the overall accuracy? This must be clearly shown.

**The authors responded to this point in the response letter but did not incorporate these reasons in the manuscript itself. This must be added to the introduction section along with the appropriate references.**

 Methods:

- The settings used for each apex locator must be clearly defined. “mid-green area on the screen” is not precise.

**For this comment, the responses of the authors are highly appreciated however, these details should be mentioned in the manuscript for accurate reproducibility of the work along with adding the references.**

- How was irrigation done? The volume used? The type of needle and gauge used? And depth of insertion of the needle? How long was the duration of irrigation?

**Again, the authors responses should be incorporated as modifications in the main text. All these details should be added for the reader to see as well.**

- Why did the authors not use EDTA? Additionally, the sequence of using EDTA and NaOCl is the most common combination of irrigating solutions used especially since working length is often verified in the middle of instrumentation procedures under the influence of mixtures of these irrigants together. It is well-known in endodontics that combinations of irrigants are often used so testing combinations of irrigants would have given higher validation of the results of this study.

**While the response of the authors to this comment is not convincing since they could have used different groups with and without EDTA, this justification or rationalization of why they did NOT use EDTA should be mentioned in the discussion section.**

- There is a major concern that the same samples were used for all the different variables. Other studies separated the entire sample into groups to avoid the influence of all of the intermediate procedures on the measurements taken last. How do the authors justify this? It would have been better to divide the sample into sub-groups for each tested variable especially since each sample represents its own control and this division of the samples would not have influenced the results, on the contrary would have enhanced the precision of the results.

**The same as above, this explanation should be added to the discussion section.**

 Results:

- The tables and figures demonstrate that the values measured are the working length values in mm however, this is impossible since the values are even in the negative? So what do these values represent? Do they represent the values registered on the apex locator screens according to the pre-determined settings? I.e. the number registered on the screen when the device indicates that this is the CORRECT working length? If so these values are quite misleading. Also, how is it possible that the ACTUAL working length is 0.087+-0.445? The authors should record the entire working length in mm from the coronal reference point to the apical constriction minus 0.5 mm as they mentioned in the methodology.

**Again, the authors must clearly show or describe that the values are the DIFFERENCE in readings and that they were recorded as either + or -.**

- The frequency of the difference between the actual working length and that measured in each condition by the two apex locators should be shown as this is the true measure that would truly demonstrate which technique was of higher accuracy in determining the WL.
- Check:
  Ramezani, M.; Bolbolian, M.; Aliakbari, M.; Alizadeh, A.; Tofangchiha, M.; Faegh, S.M.; Patini, R.; D’Amato, G. Accuracy of Three Types of Apex Locators versus Digital Periapical Radiography for Working Length Determination in Maxillary Premolars: An In Vitro Study. *Clin. Pract.* **2022**, *12*, 1043-1053.
  Kaufman AY, Keila S, Yoshpe M. Accuracy of a new apex locator: an in vitro study. Int Endod J. 2002 Feb;35(2):186-92. 

**The authors respond with a table but it was NOT included in the revised manuscript. Please add this table.**

- The choice of 5.25% NaOCl should be justified. It would have been more practical to use 2.5% as this is the most commonly used concentration clinically.

**Kindly add justification in the discussion section.**

- It is recommended that diagnostic accuracy of the apex locators in presence of the different variables be recorded in terms of specificity and sensitivity as compared to the actual working length measured. This information would be a better guide for the clinician and as such authors can make a recommendation regarding the most “accurate” apex locator to use and in association with which conditions.

**I appreciate the authors’ attempts by including this table which again should be added to the main revised text however, this is not what is meant by specificity and sensitivity. While I understand that this may be laborious to measure now, it should again be mentioned in the discussion section that diagnostic accuracy studies usually require these two measures to make a valuable deduction of clinical significance.**

- The results section should include a table for the demographic data of the sample including mean and SD of the types of teeth included, lengths of the teeth, number of canals (the authors mention single rooted but not the number of canals nor canal configurations), age of patients if present, gender….etc.

**Perhaps age and gender might not be available (and this should be mentioned in the text for the reasons the authors mentioned about anonymity of samples) however, the type of teeth, lengths, number of canals….etc should all be easy to demonstrate and should be included.**

 Discussion:

- The discussion is well-written but the authors should show the clinical significance of the results as previously commented placed in the context of the multiple variables that are present in the clinical situation. The authors showed based on the results of their study that the type of irrigating solution has an influence on measuring WL using these two apex locators? So what do the authors recommend? A particular sequence to use? A specific irrigant to use only during WL determination? In order to give value to results from laboratory studies their clinical significance must be shown.

**The response to this comment again should be included in the discussion section.**

- There should also be a section on the limitations of the study such as the limitation of performing the study on single rooted teeth, the influence of curvatures, the influence of combining different irrigants, influence of embedding medium, volume of irrigant and other limitations that may influence the extrapolation of the results in a clinical sense. Especially since most of these points were not mentioned in the discussion and it is advisable to do so.

**Thank you for adding a limitations section**

Is the work clearly and accurately presented and does it cite the current literature?

Partly

If applicable, is the statistical analysis and its interpretation appropriate?

Partly

Are all the source data underlying the results available to ensure full reproducibility?

No source data required

Is the study design appropriate and is the work technically sound?

Yes

Are the conclusions drawn adequately supported by the results?

Yes

Are sufficient details of methods and analysis provided to allow replication by others?

Partly

Reviewer Expertise:

NA

I confirm that I have read this submission and believe that I have an appropriate level of expertise to confirm that it is of an acceptable scientific standard, however I have significant reservations, as outlined above.

## References

[1] : Accuracy of Three Types of Apex Locators versus Digital Periapical Radiography for Working Length Determination in Maxillary Premolars: An In Vitro Study. *Clin Pract* .2022;12(6) : 10.3390/clinpract12060107 1043-1053 10.3390/clinpract12060107 36547115PMC9777103

[2] : Accuracy of a new apex locator: an in vitro study. *Int Endod J* .2002;35(2) : 10.1046/j.1365-2591.2002.00468.x 186-92 10.1046/j.1365-2591.2002.00468.x 11843975

# Response

**Published:** 2023-10-15

**Authors:** Shimaa Rifaat

**Affiliations:** Restorative Dental Sciences, Imam Abdulrahman Bin Faisal University, Dammam, Eastern Province, Saudi Arabia

**Reviewer (2) comments and authors’ responses:**

 In general:

 The authors have attempted to address the previous comments however, some changes still need to be made for the manuscript to be suitable for publication. All the responses to the comments should be clearly highlighted in the main manuscript file AND mentioned in the responses letter especially the page no. and line no. where these corrections were made.

***New comments are bolded.***

 Introduction:

- The rationale of the study is unclear. Coronal pre-flaring is a routine part of endodontic instrumentation and the irrigants used in this study are routinely used in everyday clinical practice in association with apex locators, so why are the authors assuming that these variables will affect the overall accuracy? This must be clearly shown.

**In updated Version 3, Page: 5, Line: 48-54**

**The authors responded to this point in the response letter but did not incorporate these reasons in the manuscript itself. This must be added to the introduction section along with the appropriate references.**

 Methods:

- The settings used for each apex locator must be clearly defined. “mid-green area on the screen” is not precise.

**In updated Version 3, Page: 7, line: 134-148**

**For this comment, the responses of the authors are highly appreciated however, these details should be mentioned in the manuscript for accurate reproducibility of the work along with adding the references.**

- How was irrigation done? The volume used? The type of needle and gauge used? And depth of insertion of the needle? How long was the duration of irrigation?

**In updated Version 3, Page: 6, Line: 120-125**

**Again, the authors responses should be incorporated as modifications in the main text. All these details should be added for the reader to see as well.**

- Why did the authors not use EDTA? Additionally, the sequence of using EDTA and NaOCl is the most common combination of irrigating solutions used especially since working length is often verified in the middle of instrumentation procedures under the influence of mixtures of these irrigants together. It is well-known in endodontics that combinations of irrigants are often used so testing combinations of irrigants would have given higher validation of the results of this study.

**While the response of the authors to this comment is not convincing since they could have used different groups with and without EDTA, this justification or rationalization of why they did NOT use EDTA should be mentioned in the discussion section.**

**Thank you for your comment. At the first steps of WL determination, we use NaOCl for removal of organic debris inside the canal without doing any cleaning and shaping with no formation of smear layer inside the canal to use any chelating agent. Hence, At the step of WL determination usually EDTA wasn’t yet introduced. Furthermore, as per the literature discussing the accuracy of WL using EAL most of the studies that showed accurate WL was with NaOCl and CHX.**

**In updated Version 3, Page: 14-15, Line: 372-376**

- There is a major concern that the same samples were used for all the different variables. Other studies separated the entire sample into groups to avoid the influence of all of the intermediate procedures on the measurements taken last. How do the authors justify this? It would have been better to divide the sample into sub-groups for each tested variable especially since each sample represents its own control and this division of the samples would not have influenced the results, on the contrary would have enhanced the precision of the results.

**The same as above, this explanation should be added to the discussion section.**

**In updated Version 3, Page: 7-8, Line: 167-170**

 Results:

- The tables and figures demonstrate that the values measured are the working length values in mm however, this is impossible since the values are even in the negative? So what do these values represent? Do they represent the values registered on the apex locator screens according to the pre-determined settings? I.e. the number registered on the screen when the device indicates that this is the CORRECT working length? If so these values are quite misleading. Also, how is it possible that the ACTUAL working length is 0.087+-0.445? The authors should record the entire working length in mm from the coronal reference point to the apical constriction minus 0.5 mm as they mentioned in the methodology.

**Again, the authors must clearly show or describe that the values are the DIFFERENCE in readings and that they were recorded as either + or -.**

**Add it in the discussion section (corrected in the statistical analysis, results, and the tables labels)**

**In updated Version 3, Page: 8-11, Line: 173-257**

- The frequency of the difference between the actual working length and that measured in each condition by the two apex locators should be shown as this is the true measure that would truly demonstrate which technique was of higher accuracy in determining the WL.
- Check:
  Ramezani, M.; Bolbolian, M.; Aliakbari, M.; Alizadeh, A.; Tofangchiha, M.; Faegh, S.M.; Patini, R.; D’Amato, G. Accuracy of Three Types of Apex Locators versus Digital Periapical Radiography for Working Length Determination in Maxillary Premolars: An In Vitro Study. *Clin. Pract.* **2022**, *12*, 1043-1053.
  Kaufman AY, Keila S, Yoshpe M. Accuracy of a new apex locator: an in vitro study. Int Endod J. 2002 Feb;35(2):186-92. 

**The authors respond with a table but it was NOT included in the revised manuscript. Please add this table.**

**In updated Version 3, Page: 12, Line: 261-280**

- The choice of 5.25% NaOCl should be justified. It would have been more practical to use 2.5% as this is the most commonly used concentration clinically.

**Kindly add justification in the discussion section.**

**In updated Version 3, Page: 13, Line: 308-309**

- It is recommended that diagnostic accuracy of the apex locators in presence of the different variables be recorded in terms of specificity and sensitivity as compared to the actual working length measured. This information would be a better guide for the clinician and as such authors can make a recommendation regarding the most “accurate” apex locator to use and in association with which conditions.

**I appreciate the authors’ attempts by including this table which again should be added to the main revised text however, this is not what is meant by specificity and sensitivity. While I understand that this may be laborious to measure now, it should again be mentioned in the discussion section that diagnostic accuracy studies usually require these two measures to make a valuable deduction of clinical significance.**

**In updated Version 3, Page: 15, Line: 382-383**

- The results section should include a table for the demographic data of the sample including mean and SD of the types of teeth included, lengths of the teeth, number of canals (the authors mention single rooted but not the number of canals nor canal configurations), age of patients if present, gender….etc.

**Perhaps age and gender might not be available (and this should be mentioned in the text for the reasons the authors mentioned about anonymity of samples) however, the type of teeth, lengths, number of canals….etc should all be easy to demonstrate and should be included.\**

**All the data were collected and tabulated and uploaded on the link bellow:**

**Rifaat S: The Effect of Coronal Pre-flaring and Root Canal Irrigant on Apex Locators Accuracy: In-Vitro Study. [Dataset]. *figshare.* 2023. 10.6084/m9.figshare.22492354.v4**

**In updated Version 3, Page: 5, Line: 82**

 Discussion:

- The discussion is well-written but the authors should show the clinical significance of the results as previously commented placed in the context of the multiple variables that are present in the clinical situation. The authors showed based on the results of their study that the type of irrigating solution has an influence on measuring WL using these two apex locators? So what do the authors recommend? A particular sequence to use? A specific irrigant to use only during WL determination? In order to give value to results from laboratory studies their clinical significance must be shown.

**In updated Version 3,** **Page: 15, Line: 396-400**

**The response to this comment again should be included in the discussion section.**

- There should also be a section on the limitations of the study such as the limitation of performing the study on single rooted teeth, the influence of curvatures, the influence of combining different irrigants, influence of embedding medium, volume of irrigant and other limitations that may influence the extrapolation of the results in a clinical sense. Especially since most of these points were not mentioned in the discussion and it is advisable to do so.

**Thank you for adding limitations section**

**References**

 1. Ramezani M, Bolbolian M, Aliakbari M, Alizadeh A, et al.: Accuracy of Three Types of Apex Locators versus Digital Periapical Radiography for Working Length Determination in Maxillary Premolars: An In Vitro Study. *Clin Pract*. 2022; **12** (6): 1043-1053 PubMed Abstract | Publisher Full Text

 2. Kaufman AY, Keila S, Yoshpe M: Accuracy of a new apex locator: an in vitro study. *Int Endod J*. 2002; **35** (2): 186-92 PubMed Abstract | Publisher Full Tex

# Reviewer report: Reviewer response for version 2

**DOI:** 10.5256/f1000research.153312.r203785

**Published:** 2023-09-19

**Authors:** Amira Salem

**Affiliations:** 1 Sir John Walsh Research Institute, Faculty of Dentistry, University of Otago, Dunedin, Otago, New Zealand; 2 Blis Technologies Limited, Dunedin, New Zealand

The Revised version of the manuscript has been improved greatly from the first version, and many comments and confusions has been addressed and clarified.

 However, there is still few comments I would like to add:

 1- In the abstract: "The significant results were shown ..." word significant only doesn't indicate whether it is significantly accurate or significantly inaccurate. although it is understood from the results later in the article, it is better to make it more clear.

 2-Hypothesis: the wording of the hypothesis can be improved, not in a past tense statement?! and more clearer

 3-Methods: a repetition in the following sentence " Using freshly mixed alginate, each tooth was mounted at the cemento-enamel junction (CEJ) level using freshly mixed alginate.

 4- Results:

 a -"Moreover, the dry medium showed t he most accurate WL with the Raypex 6, the NaOCl demonstrated t he most accurate WL with the Raypex 6 "  this is very confusing !

 b- The authors indicated that the outliers was adjusted in the results. Does the authors think that this is an error during the readings? how can this be clinically avoided ?

 c- the standard deviations in all the readings are quite high ranging from ( +/- 0.4 to 0.7) difference from the accurate Working length this gives a low general confidence on the accuracy and significance of the difference between either the irrigants used or the type of the apex locator. The authors can reflect on this in their discussion and the limitations of solely relying on their use for determining the accurate working length.

 d- "Figures 2 and 3 present the pre-flaring WL measurements for Root ZX and Raypex 6 for all irrigating solutions. The median lengths for dry medium and NaOCl were almost the same, whereas the median length for CHX significantly decreased and was closer to the actual WL.

 This was only observed with RoothZX only ? i.e figure 2 only.

 e- "Moreover, the dry medium displayed the most accurate WL with Raypex 6, f ollowed by NaOCl achieved the most accurate WL with Root ZX, and CHX showed the most accurate WL with Root ZX in all coronal pre-flaring conditions  This is also very confusing.

 f-"Figure 5 illustrates a similar pattern for the Raypax 6 apex locator"

 They are not similar; the dry and chx are similar but different from the sodium hypochlorite!

 5- Discussion:

 “When NaOCl was used as an irrigant.." In this paragraph an emphasis on "no flaring" as an advantage of accuracy with the Raypex 6 and sodium hypochlorite while the authors have already described in the previous paragraph and in table 3 that no flaring was better among all irrigants and both EAL used. It is statistically significant in this condition but not an exceptional from other observations!

 6- Conclusion:

 "In conclusion, the study results suggest that adherence to the endodontic principles of conventional access opening, coronal pre-flaring….. " However, the results  of this study indicated that the determination of WL will be more accurate before pre-flaring even though it might be necessary for other aspects of the successful RCT procedure. This is not reflected in the conclusion.!

Is the work clearly and accurately presented and does it cite the current literature?

Yes

If applicable, is the statistical analysis and its interpretation appropriate?

Partly

Are all the source data underlying the results available to ensure full reproducibility?

No source data required

Is the study design appropriate and is the work technically sound?

Yes

Are the conclusions drawn adequately supported by the results?

Partly

Are sufficient details of methods and analysis provided to allow replication by others?

Partly

Reviewer Expertise:

Endodontics, microbiology, molecular biology, biotechnology, bioengineering, product development, drug delivery

I confirm that I have read this submission and believe that I have an appropriate level of expertise to confirm that it is of an acceptable scientific standard, however I have significant reservations, as outlined above.

# Response

**Published:** 2023-10-15

**Authors:** Shimaa Rifaat

**Affiliations:** Restorative Dental Sciences, Imam Abdulrahman Bin Faisal University, Dammam, Eastern Province, Saudi Arabia

The Revised version of the manuscript has been improved greatly from the first version, and many comments and confusions has been addressed and clarified.

 However, there is still few comments I would like to add:

 1- In the abstract: "The significant results were shown ..." word significant only doesn't indicate whether it is significantly accurate or significantly inaccurate. although it is understood from the results later in the article, it is better to make it more clear.

**Thank you for the comment. This was adjusted in V3 Abstract** **for your kind review.**

 2-Hypothesis: the wording of the hypothesis can be improved, not in a past tense statement?! and more clearer

**Thank you for the comment. This was adjusted in V3, Page: 6, Line: 62.**

 3-Methods: a repetition in the following sentence " Using freshly mixed alginate, each tooth was mounted at the cemento-enamel junction (CEJ) level using freshly mixed alginate.

**Thank you for the comment. The repeated sentence has been removed.**

 4- Results:

 a -"Moreover, the dry medium showed t he most accurate WL with the Raypex 6, the NaOCl demonstrated t he most accurate WL with the Raypex 6 "  this is very confusing !

**With Raypex 6 the most accurate results were shown with dry medium, NaOCl irrigant, and CHX respectively in all pre-flaring conditions.**

 b- The authors indicated that the outliers was adjusted in the results. Does the authors think that this is an error during the readings? how can this be clinically avoided?

**The authors did not attribute the presence of any reading errors, but acknowledged that outliers may arise from various causes, including samples measurement and samples anatomy variations. Therefore, avoiding the outliers clinically could be achieved by using calibrated instruments for precision readings, training operators to use instruments correctly, adhering to standardized data collection procedures, carefully verifying data for errors prior to analysis, and employing statistical methods to detect and eliminate outliers resulting from measurement errors. These measures can lower the likelihood of measurement-induced outliers and enhance result accuracy.**

 c- the standard deviations in all the readings are quite high ranging from ( +/- 0.4 to 0.7) difference from the accurate Working length this gives a low general confidence on the accuracy and significance of the difference between either the irrigants used or the type of the apex locator. The authors can reflect on this in their discussion and the limitations of solely relying on their use for determining the accurate working length.

**The high standard deviations in the measurements indicate a considerable degree of variability in the data. This variability can be attributed to a range of factors, such as the inherent limitations of the apex locators, or potential errors in the experimental setup** **.**

**The results of the study indicate that EALs devices may not consistently provide precise measurements, as suggested by the wide standard deviations. EALs are valuable tools in root canal measurement, they should not be the sole basis for determining working length, and other complementary methods or clinical judgment should be used to enhance accuracy and reliability.**

 d- "Figures 2 and 3 present the pre-flaring WL measurements for Root ZX and Raypex 6 for all irrigating solutions. The median lengths for dry medium and NaOCl were almost the same, whereas the median length for CHX significantly decreased and was closer to the actual WL.

 This was only observed with RoothZX only ? i.e figure 2 only.

**Yes. This was observed for CHX irrigant when using Root ZX.**

 e- "Moreover, the dry medium displayed the most accurate WL with Raypex 6, f ollowed by NaOCl achieved the most accurate WL with Root ZX, and CHX showed the most accurate WL with Root ZX in all coronal pre-flaring conditions. This is also very confusing.

**With Raypex 6 the most accurate results were shown with dry medium, NaOCl irrigant, and CHX respectively in all pre-flaring conditions.** **Whereas Root ZX showed the most accurate results with CHX irrigant, dry medium, and NaOCl respectively in all coronal pre-flaring conditions.**

 f-"Figure 5 illustrates a similar pattern for the Raypax 6 apex locator"

 They are not similar; the dry and chx are similar but different from the sodium hypochlorite!

**Adjusted in updated V3 manuscript. Page:10, Line: 232-233.**

 5-Discussion:

 “When NaOCl was used as an irrigant.." In this paragraph an emphasis on "no flaring" as an advantage of accuracy with the Raypex 6 and sodium hypochlorite while the authors have already described in the previous paragraph and in table 3 that no flaring was better among all irrigants and both EAL used. It is statistically significant in this condition but not an exceptional from other observations!

**It has been demonstrated from previous studies that performing coronal pre-flaring has a substantial impact on enhancing the precision of measuring the working length of molars' root canals. This improvement is not solely attributed to better access but also to the reduction in file resistance, which, in turn, facilitates the smooth insertion of files into the root canal towards the tooth's apex. Additionally, it promotes improved circulation of irrigation within the root canal, thereby decreasing the likelihood of debris being pushed out towards the apex and reducing the risk of instrument breakage. It's worth noting that while previous studies utilized rotary files for canal preparation, the present study employed Gates Glidden instruments.**

**(Discussed in the Introduction V3 Page:** **5, Line: 48-54)**

 6- Conclusion:

 "In conclusion, the study results suggest that adherence to the endodontic principles of conventional access opening, coronal pre-flaring….. " However, the results  of this study indicated that the determination of WL will be more accurate before pre-flaring even though it might be necessary for other aspects of the successful RCT procedure. This is not reflected in the conclusion.!

**The study's findings emphasize that sticking to established endodontic principles like conventional access opening, coronal pre-flaring, and maintaining patency are crucial for obtaining precise and consistent measurements of the working length (WL) with Root ZX and Raypex 6 electronic apex locators (EALs). However, the effect of coronal pre-flaring wasn't clear due to the use of Gates Glidden instruments instead of rotary instruments that showed better results.**

**Page: 13, Line: 302-307**

# Reviewer report: Reviewer response for version 1

**DOI:** 10.5256/f1000research.146267.r174824

**Published:** 2023-06-19

**Authors:** Rania El Backly

**Affiliations:** 1 Endodontics, Conservative Dentistry Department and Tissue Engineering Laboratories, Faculty of Dentistry, Alexandria University, Alexandria, Alexandria Governorate, Egypt

In general:

 The manuscript addresses an important topic relevant to the everyday practice of endodontics. However, it requires substantial language editing and rewriting to be more concise and focused. It is recommended to follow the PRILE 2021 guidelines for documenting laboratory studies in endodontics.

 Title:

 I suggest modification of the title to be as follows:

 The influence of coronal pre-flaring and type of root canal irrigant on accuracy of working length determination using electronic apex locators

 Abstract:

 The abstract needs to be re-written to clearly demonstrate the specific objective of the study as well as the hypothesis. The methodology is unclear and non-specific. The groups are not clear. Results must be mentioned to clearly show quantitative data and show the differences between the groups used. Again, the conclusion must be more specific and clearly reflect the specific outcomes of this study.

 Introduction:

- The authors are advised to add a hypothesis statement at the end of the introduction section.
- The rationale of the study is unclear. Coronal pre-flaring is a routine part of endodontic instrumentation and the irrigants used in this study are routinely used in everyday clinical practice in association with apex locators, so why are the authors assuming that these variables will affect the overall accuracy? This must be clearly shown.

Methods:

- Sample size calculation: The exact number of teeth per group must be mentioned.
- Sample selection and sample preparation sections contain repeated information.
- The grouping is not clear. Upon reading the methodology it seems that the 120 teeth were subjected to all conditions of measurements? This must be clearly shown in a table and a flow chart should be included to show the exact procedure.
- The experimental set-up needs to be more accurately described.
- The settings used for each apex locator must be clearly defined. “mid-green area on the screen” is not precise.
- How was irrigation done? The volume used? The type of needle and gauge used? And depth of insertion of the needle?
- How long was the duration of irrigation?
- Why did the authors not use EDTA? Additionally, the sequence of using EDTA and NaOCl is the most common combination of irrigating solutions used especially since working length is often verified in the middle of instrumentation procedures under the influence of mixtures of these irrigants together. It is well-known in endodontics that combinations of irrigants are often used so testing combinations of irrigants would have given higher validation of the results of this study.
- There is a major concern that the same samples were used for all the different variables. Other studies separated the entire sample into groups to avoid the influence of all of the intermediate procedures on the measurements taken last. How do the authors justify this? It would have been better to divide the sample into sub-groups for each tested variable especially since each sample represents its own control and this division of the samples would not have influenced the results, on the contrary would have enhanced the precision of the results.

Results:

- The tables and figures demonstrate that the values measured are the working length values in mm however, this is impossible since the values are even in the negative? So what do these values represent? Do they represent the values registered on the apex locator screens according to the pre-determined settings? I.e. the number registered on the screen when the device indicates that this is the CORRECT working length? If so these values are quite misleading. Also, how is it possible that the ACTUAL working length is 0.087+-0.445? The authors should record the entire working length in mm from the coronal reference point to the apical constriction minus 0.5 mm as they mentioned in the methodology.
- The frequency of the difference between the actual working length and that measured in each condition by the two apex locators should be shown as this is the true measure that would truly demonstrate which technique was of higher accuracy in determining the WL.
- Check:
  -  Ramezani, M.; Bolbolian, M.; Aliakbari, M.; Alizadeh, A.; Tofangchiha, M.; Faegh, S.M.; Patini, R.; D’Amato, G. Accuracy of Three Types of Apex Locators versus Digital Periapical Radiography for Working Length Determination in Maxillary Premolars: An In Vitro Study. *Clin. Pract.* **2022**, *12*, 1043-1053.
  - Kaufman AY, Keila S, Yoshpe M. Accuracy of a new apex locator: an in vitro study. Int Endod J. 2002 Feb;35(2):186-92. 
- The choice of 5.25% NaOCl should be justified. It would have been more practical to use 2.5% as this is the most commonly used concentration clinically.
- It is recommended that diagnostic accuracy of the apex locators in presence of the different variables be recorded in terms of specificity and sensitivity as compared to the actual working length measured. This information would be a better guide for the clinician and as such authors can make a recommendation regarding the most “accurate” apex locator to use and in association with which conditions.
- The results section should include a table for the demographic data of the sample including mean and SD of the types of teeth included, lengths of the teeth, number of canals (the authors mention single rooted but not the number of canals nor canal configurations), age of patients if present, gender….etc.

Discussion:

- The discussion is well-written but the authors should show the clinical significance of the results as previously commented placed in the context of the multiple variables that are present in the clinical situation. The authors showed based on the results of their study that the type of irrigating solution has an influence on measuring WL using these two apex locators? So what do the authors recommend? A particular sequence to use? A specific irrigant to use only during WL determination? In order to give value to results from laboratory studies their clinical significance must be shown.
- There should also be a section on the limitations of the study such as the limitation of performing the study on single rooted teeth, the influence of curvatures, the influence of combining different irrigants, influence of embedding medium, volume of irrigant and other limitations that may influence the extrapolation of the results in a clinical sense. Especially since most of these points were not mentioned in the discussion and it is advisable to do so. Additionally, a statement of accepting or rejecting the null hypothesis which should be mentioned in the introduction section is recommended.

Is the work clearly and accurately presented and does it cite the current literature?

Partly

If applicable, is the statistical analysis and its interpretation appropriate?

Partly

Are all the source data underlying the results available to ensure full reproducibility?

No source data required

Is the study design appropriate and is the work technically sound?

Yes

Are the conclusions drawn adequately supported by the results?

Yes

Are sufficient details of methods and analysis provided to allow replication by others?

Partly

Reviewer Expertise:

Endodontics, regenerative endodontics, tissue engineering, regenerative medicine

I confirm that I have read this submission and believe that I have an appropriate level of expertise to confirm that it is of an acceptable scientific standard, however I have significant reservations, as outlined above.

## References

[1] : Accuracy of Three Types of Apex Locators versus Digital Periapical Radiography for Working Length Determination in Maxillary Premolars: An In Vitro Study. *Clin Pract* .2022;12(6) : 10.3390/clinpract12060107 1043-1053 10.3390/clinpract12060107 36547115PMC9777103

[2] : Accuracy of a new apex locator: an in vitro study. *Int Endod J* .2002;35(2) : 10.1046/j.1365-2591.2002.00468.x 186-92 10.1046/j.1365-2591.2002.00468.x 11843975

# Response

**Published:** 2023-07-03

**Authors:** Shimaa Rifaat

**Affiliations:** Restorative Dental Sciences, Imam Abdulrahman Bin Faisal University, Dammam, Eastern Province, Saudi Arabia

**Dear reviewer (2), (Dr. Rania Al Backly)**

**Thank you very much for your valuable comments. They are a great addition to our research. Regarding the mentioned points, kindly check below for further details.**

1) In general:

The manuscript addresses an important topic relevant to the everyday practice of endodontics. However, it requires substantial language editing and rewriting to be more concise and focused. It is recommended to follow the PRILE 2021 guidelines for documenting laboratory studies in endodontics.

 Thank you for your comment and further editing and rewriting will be done in the updated version to make it clear for the readers.

2) Title:  I suggest modification of the title to be as follows:

The influence of coronal pre-flaring and type of root canal irrigant on accuracy of working length determination using electronic apex locators.

 Thank you for your comment. And I suggest modifying it too to be:

 “The effect of coronal pre-flaring and type of root canal irrigant on working length accuracy using electronic apex locators.”

3) Abstract:

The abstract needs to be re-written to clearly demonstrate the specific objective of the study as well as the hypothesis. The methodology is unclear and non-specific. The groups are not clear. Results must be mentioned to clearly show quantitative data and show the differences between the groups used. Again, the conclusion must be more specific and clearly reflect the specific outcomes of this study.

 Updated Abstract:

 Background: The success of root canal treatment relies on achieving proper root canal preparation and precise root canal filling, including reaching the full working length up to the apical constriction. Electronic apex locators (EALs) have been introduced to enhance the accuracy of working length determination. However, the optimal irrigation protocol for each generation of EALs remains unclear

 Hypothesis: No significant difference in the accuracy of working length determination would be detected by coronal pre-flaring and the type of irrigant, varying with different generations of EALs

 Methods: Actual working lengths of 120 single-rooted human teeth were measured and compared with working lengths determined by the 3 ^rd^ generation EAL (Root ZX) and 6 ^th^ generation EAL (Raypex 6). Measurements were taken in a dry medium, in the presence of 5.25% sodium hypochlorite, and 2% chlorhexidine. The teeth were evaluated both without coronal pre-flaring and after coronal pre-flaring using the same irrigating solutions. Data were collected, tabulated, and analyzed using one-way ANOVA with post-hoc tests to assess significant differences in average working length accuracy between the actual working length, Root ZX, and Raypex 6 EALs

 Results: The results of the current study showed the best results with the Raypex 6 apex locator were in dry medium in all conditions of pre-flaring (without pre-flaring & with pre-faring) (T=3.8, P=0.001) and (T=5.4, P=0.001) respectively. Root ZX apex locator achieved significant results with the CHX in all conditions of pre-flaring (without pre-flaring & with pre-faring): (T=3.85, P=0.001) and (T=3.99, P=0.001) respectively

 Conclusion: The study found that the Raypex 6 electronic apex locator (EAL) provided significantly more accurate readings closer to the actual working length compared to the Root ZX EAL. The best results with the Raypex 6 were obtained when measurements were taken in a dry medium, in all the pre-flaring conditions. The Root ZX EAL achieved significant results with the use of chlorhexidine (CHX) as an irrigant in all the pre-flaring conditions.

4) Introduction:

A) The authors are advised to add a hypothesis statement at the end of the introduction section.

 Hypothesis: No significant difference in the accuracy of working length determination would be detected by coronal pre-flaring and the type of irrigant, varying with different generations of EALs.

B) The rationale of the study is unclear. Coronal pre-flaring is a routine part of endodontic instrumentation and the irrigants used in this study are routinely used in everyday clinical practice in association with apex locators, so why are the authors assuming that these variables will affect the overall accuracy? This must be clearly shown.

 Thank you for your question. It is clear nowadays that some research studies recommended the Contracted endodontic cavities (CECs) that were developed from the concept of minimally invasive dentistry and provide an alternative to traditional endodontic cavities (TECs).  They claimed that the CECs preserve the mechanical stability of teeth and preserve more of the dentin as discussed by Lovisi et al 2018, Weiqi et al 2022, Wang et al 2023. That’s why the working length determination without coronal flaring was used in this study as it is a way some researchers recommended and supported by some of them.

*Alovisi M, Pasqualini D, Musso E, Bobbio E, Giuliano C, Mancino D, Scotti N, Berutti E. Influence of contracted endodontic access on root canal geometry: an in vitro study. Journal of endodontics. 2018 Apr 1;44(4):614-20.*

*Weiqi Peng, Xuedong Zhou, Yuan Gao, Xin Xu, Effect of Access Cavity Preparation on Dentin Preservation, Biomechanical Property, and Instrumentation Efficacy: A Micro–Computed Tomographic Study, Journal of Endodontics, Volume 48, Issue 5,2022, 659-668, ISSN 0099-2399, https://doi.org/10.1016/j.joen.2021.12.012*

*Wang, X., Wang, D., Wang, Yr. et al. Effect of access cavities on the biomechanics of mandibular molars: a finite element analysis. BMC Oral Health **23**, 196 (2023). https://doi.org/10.1186/s12903-023-02878-3*

 In addition, the effect of the irrigant on the reading accuracy and the electrical conductivity of various irrigant used and how it affects the accuracy of electronic apex locators. This will work hand in hand with the most accurate determination of the WL. Therefore, it would ensure the root canal treatment outcome and prognosis.

5) Methods:

A) Sample size calculation: The exact number of teeth per group must be mentioned.

 120 teeth were used. All the sample size experienced all the preparation scenarios and under all conditions to minimize the variables for among all the variables for each sample.

B) Sample selection and sample preparation sections contain repeated information.

 Thank you for your comment. It will be revised and adjusted in the updated version.

C) The grouping is not clear. Upon reading the methodology it seems that the 120 teeth were subjected to all conditions of measurements. This must be clearly shown in a table and a flow chart should be included to show the exact procedure.

 Yes, the 120 teeth were subjected to all conditions of measurements. This is supplied in the flow chart linked to the original manuscript on a figshare link.

 For your kind review: *Rifaat S: The Effect of Coronal Pre-flaring and Root Canal Irrigant on Apex Locators Accuracy: In-Vitro Study. [Dataset]. figshare. 2023. 10.6084/m9.figshare.22492354.v4*

D) The experimental set-up needs to be more accurately described.

 Thank you for your comment. It will be described clearly in the updated version.

E) The settings used for each apex locator must be clearly defined. “mid-green area on the screen” is not precise.

 For Root ZX, working length determination was established at the point when the screen displays the line just in the middle of apex and 1 mark which corresponds to 0.5 mm short of radiologic apex. For Raypex 6, the third green line, just before the yellow lines, which corresponds to 0.5 mm short of radiographic apex was determined for working length. The measures were recorded as electronic measurement (EM) if they were stable for at least 5 seconds as described by Aydin et al 2015.

 Moreover, When the file tip reaches or is close to the apical constriction, the apex locator's screen display typically indicates this by showing the file position within the mid-green area. Moreover, both Root ZX and Raypex 6 EALs also have an audio indicator that emits a sound, such as a continuous or intermittent beep, when the file approaches or reaches the apical constriction. The change in sound indicates that the file is close to the desired working length, assisting the clinician in achieving precise measurements. Furthermore, both Root ZX and Raypex 6 feature a digital display that shows the distance between the file tip and the apical constriction in millimeters as reported by Higa et al 2009. Besides, Calibration of EALs before each use, according to the manufacturer's instructions was performed to ensure precision of WL measurements as recommended by Cinar et al 2020.

*Aydin U, Karataslioglu E, Aksoy F, Yildirim C. In vitro evaluation of Root ZX and Raypex 6 in teeth with different apical diameters. J Conserv Dent. 2015 Jan-Feb;18(1):66-9. doi: 10.4103/0972-0707.148899. PMID: 25657531; PMCID: PMC4313483”*

*Higa RA, Adorno CG, Ebrahim AK, Suda H. Distance from file tip to the major apical foramen in relation to the numeric meter reading on the display of three different electronic apex locators. Int Endod J. 2009;42(12):1065-1070. doi:10.1111/j.1365-2591.2009.01629.x”*

*“Çınar F, Üstün Y. Ex Vivo Evaluation of the Accuracy of 3 Electronic Apex Locators in Different Environments: A Micro-Computed Tomography Study. Eur Endod J. 2020 Dec;5(3):226-230. doi: 10.14744/eej.2020.30633. PMID: 33353910; PMCID: PMC7881377.”*

F) How was irrigation done? The volume used. The type of needle and gauge used. And depth of insertion of the needle?

 Irrigation was done with 23G with apical vented needle (Ultrdent Products, South Jordan, UT, USA). The needle was introduced until resistance was felt and then reduced 1 mm from the needle penetration depth to prevent obliteration of the root canal lumen as illustrated by Guerreiro et al 2013. 3 ml of 5.25% NaOCl and 2% CHX were used alternatively in the presence and absence of coronal pre-flaring as used by Spoorthy et al 2013.

*Guerreiro-Tanomaru JM, Loiola LE, Morgental RD, Leonardo RD, Tanomaru-Filho M. Efficacy of four irrigation needles in cleaning the apical third of root canals. Brazilian Dental Journal. 2013;24:21-4.*

*Spoorthy E, Velmurugan N, Ballal S, Nandini S. Comparison of irrigant penetration up to working length and into simulated lateral canals using various irrigating techniques. International endodontic journal. 2013 Sep;46(9):815-22.*

G) How long was the duration of irrigation?

 It was for 2 minutes as suggested by Fernandes et al 2021.

*Fernandes AL, da Silva Limoeiro AG, Kato AS, Pelegrine RA, de Martin AS, Rocha DG, da Silveira Bueno CE. Analysis of two irrigation methods in root canal disinfection against E. Faecalis biofilm under the influence of the concentration, volumen, and time in contact of the irrigant. Research, Society and Development. 2021 Jul 13;10(8):e32610817478.*

H) Why did the authors not use EDTA? Additionally, the sequence of using EDTA and NaOCl is the most common combination of irrigating solutions used especially since working length is often verified in the middle of instrumentation procedures under the influence of mixtures of these irrigants together. It is well-known in endodontics that combinations of irrigants are often used so testing combinations of irrigants would have given higher validation of the results of this study.

 Thank you for your valuable comment. In our research we preferred to use only one irrigant sequence to prevent the results bias. As if this recommended protocol used, the authors will not be able which irrigant electroconductivity is higher: the NaOCl or the EDTA. But as we have a benchmark after our study results, we can consider using combinations with the NaOCl in the future to evaluate their accuracy.

I) There is a major concern that the same samples were used for all the different variables. Other studies separated the entire sample into groups to avoid the influence of all of the intermediate procedures on the measurements taken last. How do the authors justify this? It would have been better to divide the sample into sub-groups for each tested variable especially since each sample represents its own control and this division of the samples would not have influenced the results, on the contrary would have enhanced the precision of the results.

 Thank you for the nice comment. We agree with you. But we tried to eliminate any variable that could take place among each group. That’s why we subjected each tooth was its same anatomical variation, diameter, length, dentin thickness to all the study variables to be more precise while calculating the deviation from the actual working length. Because all the mentioned points above could contribute to many changes while collecting the study results and could affect the study accuracy.

6) Results:

A) The tables and figures demonstrate that the values measured are the working length values in mm however, this is impossible since the values are even in the negative? So what do these values represent? Do they represent the values registered on the apex locator screens according to the pre-determined settings? I.e. the number registered on the screen when the device indicates that this is the CORRECT working length? If so these values are quite misleading. Also, how is it possible that the ACTUAL working length is 0.087+-0.445? The authors should record the entire working length in mm from the coronal reference point to the apical constriction minus 0.5 mm as they mentioned in the methodology.

 Thank you for your comment. Regarding the readings for our results, it is the difference between the mean WL determined by the 2 calibrated evaluators and the actual working length. Considering the working length is at zero difference and we are calculating the difference or deviation from the zero either increased by (+) or decreased by (-).

B) The frequency of the difference between the actual working length and that measured in each condition by the two apex locators should be shown as this is the true measure that would truly demonstrate which technique was of higher accuracy in determining the WL.

 Thank you for your valuable comment. The below table shows the frequency of deviations from actual working length for both electronic apex locators with each irrigation medium in the absence and presence of coronal pre-flaring. The frequencies are presented in table below were calculated for the differences between the measured and the actual WL within the range of ±0.5 and greater than 1. In the absence of coronal pre-flaring the Root ZX (3 ^rd^ generation) showed more accuracy as most of the values were within the range of ±0.5 in each irrigation system, whereas Raypex 6 (6 ^th^ generation) showed less accuracy than Root ZX while having majority of the values greater than 1 which is showing the far distance from actual WL. A similar trend was observed in irrigation media used in the presence of coronal pre-flaring except in dry medium where Raypex 6 apex locator showed more accuracy with the most values near to ±0.5.

 Medium     EAL      WL deviation in ±0.5    WL deviation > 1    WL deviation in ±0.5    WL deviation > 1  

                                       No.     Freq.             No.    Freq.              No.      Freq.              No.       Freq.

 Dry      3rd generation     111      94.1            2.5     2.5                 99        83.9                 4         3.4

             6th generation     102      86.4           10.2    10.2              104       88.1               14        11.9

 NaOCl  3rd generation    104      88.1            2.5      2.5               102       86.4                7          5.9

              6th generation    100      84.7           12.7    12.7             84         71.2                32        27.1

 CHX     3rd Generation   107      90.7            4.2      4.2               114        96.6               4         3.4

             6th Generation     96       81.4           15.3     15.3             101        85.6              17       14.4

C) The choice of 5.25% NaOCl should be justified. It would have been more practical to use 2.5% as this is the most commonly used concentration clinically.

 A 2021 study by Fernandes et al 2021 showed that the 5.25% NaOCl showed a significantly greater reduction in E. faecalis than 2.5% NaOCl. Therefore, some researchers suggested the usage of the 5.25% NaOCl.

*Fernandes AL, da Silva Limoeiro AG, Kato AS, Pelegrine RA, de Martin AS, Rocha DG, da Silveira Bueno CE. Analysis of two irrigation methods in root canal disinfection against E. Faecalis biofilm under the influence of the concentration, volumen, and time in contact of the irrigant. Research, Society and Development. 2021 Jul 13;10(8):e32610817478-.*

D) It is recommended that diagnostic accuracy of the apex locators in presence of the different variables be recorded in terms of specificity and sensitivity as compared to the actual working length measured. This information would be a better guide for the clinician and as such authors can make a recommendation regarding the most “accurate” apex locator to use and in association with which conditions.

 Thank you for your comment. The table below shows the relative error for each apex locator for accuracy of measurement of WL. Root ZX was found to be the most accurate measurement tool for WL in Dry (1.57%) and CHX (1.54%) irrigation system. Whereas with NaOCL,  Raypax 6 showed high accuracy (1.76%) in teeth without coronal pre-flaring. However, in presence of pre-flaring, Root ZX showed high accuracy in NaOCL (2.21%) and CHX (1.93%) compared to Raypex 6.

 Irrigation Solution             Apex Locator                                        Relative Error

                                                                                   Without Pre-flaring         With Pre-flaring

      Dry                              3rd generation                      1.57%                             2.48%

                                          6th generation                      1.58%                             1.74%

     NaOCl                          3rd generation                      1.97%                             2.21%

                                          6th generation                      1.76%                             2.61%

     CHX                             3rd Generation                      1.54%                            1.93%

                                          6th generation                       2.03%                            2.28%

E) The results section should include a table for the demographic data of the sample including mean and SD of the types of teeth included, lengths of the teeth, number of canals (the authors mention single rooted but not the number of canals nor canal configurations), age of patients if present, gender….etc.

 Regarding the demographic data for the sample, it was selected as follows: single rooted teeth with single canals of almost same length. Regarding the age and gender of the patient’s, …. etc. We don’t have this data as teeth were collected from the disposal of IAU dental hospital where patient identification were kept anonymous even after signing in the consent.

7) Discussion:

A) The discussion is well-written, but the authors should show the clinical significance of the results as previously commented placed in the context of the multiple variables that are present in the clinical situation. The authors showed based on the results of their study that the type of irrigating solution has an influence on measuring WL using these two apex locators? So, what do the authors recommend? A particular sequence to use? A specific irrigant to use only during WL determination? To give value to results from laboratory studies their clinical significance must be shown.

 The author’s suggestions are:

 While using the 6 ^th^ generation EAL (Raypex 6), dry medium is the best way to measure the most accurate WL. On the other hand, while using the 3 ^rd^ generation EAL (Root ZX), it is better to use 2% CHX while measuring the WL to achieve the most accurate readings.

 Therefore, it is very important to know the specific irrigating medium used with each specific EAL to achieve the most accurate WL results during the root canal treatment.

B) There should also be a section on the limitations of the study such as the limitation of performing the study on single rooted teeth, the influence of curvatures, the influence of combining different irrigants, influence of embedding medium, volume of irrigant and other limitations that may influence the extrapolation of the results in a clinical sense. Especially since most of these points were not mentioned in the discussion and it is advisable to do so. Additionally, a statement accepting or rejecting the null hypothesis which should be mentioned in the introduction section is recommended.

 Thank you for your comment.

 - Regarding the limitation of the study:

 Our study was conducted only on single rooted teeth with initial file size 15 while using one type of irrigant at a time to be used as a benchmark for further studies in the future. But our study limitations may include: the usage curved canals teeth with various degrees of curvature and with bigger apical foramen sizes may affect the given results. Furthermore, the usage of one irrgant at a time results may be different. Moreover, agitation of the irrigating solution may affect the electrical conductivity and hence affects the reading accuracy of EAL. Irrigation volume, concentration, temperature, and way of application may influence the reading accuracy as well. In addition, applying the current study in-vivo in the presence of the patient’s body fluids may have an influence as well. All these points should be taken into consideration in any future studies.

 - The null hypothesis was partially rejected due to the differences in the obtained results from the different irrigating solutions when used with each EAL. While it showed no significant difference between with/without coronal flaring groups.

# Reviewer report: Reviewer response for version 1

**DOI:** 10.5256/f1000research.146267.r174821

**Published:** 2023-06-05

**Authors:** Amira Salem

**Affiliations:** 1 Sir John Walsh Research Institute, Faculty of Dentistry, University of Otago, Dunedin, Otago, New Zealand; 2 Blis Technologies Limited, Dunedin, New Zealand

The order of procedure?

 Was CHX used on the same tooth as a last testing group, if so how do you justify that the results is not because multiple file insertions and a partial debridement specially after using sodium hypochlorite solution so it is giving more accurate results with this apex locator. Was there any testing group that has CHX used immediately after the dry group?

 Table 3 label?

 Check the label: all have Root ZX label

 In all tables is the “mean” WL compared to the measured working length by microscope?

 If so this means that either the positive or negative value closer to the zero is the most accurate? Please clarify

 What are the numbers beside asterisks indicate? How many outliers were excluded from the mean measured. 

 In discussion: interpretation contradicts results “but the closest mean WL with actual WL was recorded in without coronal pre-flaring irrigation groups” comment on table 3.

 “accurate results were observed in the canals that were prepared with coronal pre-flaring before working length (WL) determination in comparison to the canals that were prepared without coronal pre-flaring”? in discussion.

 “However, it is contradicted by a previous study conducted by João Marcelo da Silva Teixeira *et al.* (2012) who concluded that the usage of Gates Glidden burs for cervical pre-flaring did not significantly influence the accuracy of the apical placement of the apex locator when determining the actual working length due to insufficient removal of coronal dentin when compared with the rotary system for preparing coronal pre-flaring” Not clear statement: was Gates Glidden burs better than rotary system or not, or there was no difference.

 “Consequently, the difference was statistically significant in the roots with coronal pre-flaring prior to working length determination when using Raybex 6 apex locator and NaOCl as an irrigant” Compared to what?

 “While comparing all the groups of Raypex 6 (with or without coronal pre-flaring) regardless of the type of irrigant used, significant results were seen when compared to the same groups of Root ZX (T=2.86, p=0.005)”. More accurate? If so, not true statement related to table 3?

 “In the case of using NaOCl as an irrigant, it showed significant results when used with Raypex 6 even without coronal pre-flaring” compared to what? To RootZX? With pre flaring it is less accurate.

 The results showed that the readings when using Raypex 6 (6 ^th^ generation electronic apex locator) are significantly closer to the actual working length than Root ZX (3 ^rd^ generation electronic apex locator) for the with/without coronal pre-flaring groups. Doesn’t match with the results of table 3.

 In accordance with our study, Root ZX apex locator achieved significant results with the CHX : maybe due to that it is the last tested irrigant and could be due to more debris and tissues removed as it is also better Root ZX in all groups for after flaring.

Is the work clearly and accurately presented and does it cite the current literature?

Yes

If applicable, is the statistical analysis and its interpretation appropriate?

Partly

Are all the source data underlying the results available to ensure full reproducibility?

No source data required

Is the study design appropriate and is the work technically sound?

Yes

Are the conclusions drawn adequately supported by the results?

Partly

Are sufficient details of methods and analysis provided to allow replication by others?

Partly

Reviewer Expertise:

Endodontics, microbiology, molecular biology, biotechnology, bioengineering, product development, drug delivery

I confirm that I have read this submission and believe that I have an appropriate level of expertise to confirm that it is of an acceptable scientific standard, however I have significant reservations, as outlined above.

# Response

**Published:** 2023-07-03

**Authors:** Shimaa Rifaat

**Affiliations:** Restorative Dental Sciences, Imam Abdulrahman Bin Faisal University, Dammam, Eastern Province, Saudi Arabia

**Dear Reviewer (1), (Dr. Amira Salem)**

**Thank you very much for your valuable comments. They are a great addition to our research. Regarding the mentioned points, kindly check below for further details.**

 1) The order of procedure? Was CHX used on the same tooth as a last testing group, if so, how do you justify that the results is not because multiple file insertions and a partial debridement specially after using sodium hypochlorite solution so it is giving more accurate results with this apex locator. Was there any testing group that has CHX used immediately after the dry group?

 Yes, CHX was used for the same tooth as the last testing group, without using the CHX immediately after the dry group.

 A) We inserted the file only once for each medium (Dry, NaOCl, and CHX).

 B) We used distilled water between each medium to wash, neutralize and stop the effect of each material used before moving to the next.

 C) NaOCl is used routinely after access opening to dissolve all the pulp tissue remnants before the step of WL determination in all samples. Afterwards, the Dry, NaOCl, or CHX media was used to check the working length in all samples while using the electronic apex locators.

 D) The materials were used as a transmitting media not a debridement agent.

 E) The minimum exposure time for maximum effectiveness for the NaOCl is 20 minutes followed by 5minutes MTAD as a final rinse to ensure the complete debridement as stated by Lotfi et al 2012. Moreover, Bonnie et al 2009 reported that the maximum effectiveness for the 5% NaOCl as an antibacterial agent is at 40 minutes usage. In addition, Chen et al 2023 found that the application of NaOCl alone could be ineffective during chemical preparation of the canal and it should be agitated either by heat, ultrasonically, or followed by the usage of other materials. This concluded that only in this situation we can use NaOCl as a debridement agent otherwise it will not be an effective debriding agent, hence, the materials used in the current study was used only as transmitting medium for WL reading accuracy while using the EALs.

*Lotfi M, Moghaddam N, Vosoughhosseini S, Zand V, Saghiri MA. Effect of Duration of Irrigation with Sodium Hypochlorite in Clinical Protocol of MTAD on Removal of Smear Layer and Creating Dentinal Erosion. J Dent Res Dent Clin Dent Prospects. 2012;6(3):79-84. doi:10.5681/joddd.2012.017*

*Bonnie Retamozo, Shahrokh Shabahang, Neal Johnson, Raydolfo M. Aprecio, Mahmoud Torabinejad, Minimum Contact Time and Concentration of Sodium Hypochlorite Required to Eliminate Enterococcus faecalis, Journal of Endodontics, Volume 36, Issue 3, 2010, 520-523, ISSN 0099-2399, https://doi.org/10.1016/j.joen.2009.12.005.*

*Chen Cai, Xuan Chen, Yang Li, Qianzhou Jiang, "Advances in the Role of Sodium Hypochlorite Irrigant in Chemical Preparation of Root Canal Treatment", BioMed Research International, vol. 2023, Article ID 8858283, 17 pages, 2023. https://doi.org/10.1155/2023/8858283.*

Table 3 label? Check the label: all have Root ZX label

 Thank you for your comment. Adjustments were done in the updated manuscript.

In all tables is the “mean” WL compared to the measured working length by microscope?

If so this means that either the positive or negative value closer to the zero is the most accurate? Please clarify.

 Yes, you are correct. The means that are either positive or negative value closer to zero are the most accurate values.

What are the numbers beside asterisks indicate? How many outliers were excluded from the mean measured.

 A) The numbers beside Asterisks are the P value that indicates the significance in the given results.

 B) The value of outliers was replaced with the median of the data. They were not deleted but adjusted.

In discussion: interpretation contradicts results “but the closest mean WL with actual WL was recorded in without coronal pre-flaring irrigation groups” comment on table 3.

“accurate results were observed in the canals that were prepared with coronal pre-flaring before working length (WL) determination in comparison to the canals that were prepared without coronal pre-flaring”? in discussion.

 Thank you for your comment. Adjustments were done in the updated manuscript.

“However, it is contradicted by a previous study conducted by João Marcelo da Silva Teixeira *et al.* (2012) who concluded that the usage of Gates Glidden burs for cervical pre-flaring did not significantly influence the accuracy of the apical placement of the apex locator when determining the actual working length due to insufficient removal of coronal dentin when compared with the rotary system for preparing coronal pre-flaring” Not clear statement: was Gates Glidden burs better than rotary system or not, or there was no difference.

 Thank you for your comment. Rephrasing the statement to make it clearer were done as follows and modified in the text. “However, a prior study conducted by João Marcelo da Silva Teixeira *et al.* (2012) reported that the usage of Gates Glidden burs for cervical pre-flaring did not significantly influence the accuracy of the apical placement of the apex locator when determining the actual working length. This may be due to the insufficient removal of coronal dentin when G.G. were used. On the contrary, the usage of rotary system for preparing coronal pre-flaring can significantly influence the WL determination.

“Consequently, the difference was statistically significant in the roots with coronal pre-flaring prior to working length determination when using Raypex 6 apex locator and NaOCl as an irrigant” Compared to what?

 Consequently, the difference was statistically significant in the roots with coronal pre-flaring prior to working length determination when using Raybex 6 apex locator and NaOCl as an irrigant (T=4.5, p=0.0001) when compared with roots without pre-flaring and using NaOCl.

“While comparing all the groups of Raypex 6 (with or without coronal pre-flaring) regardless of the type of irrigant used, significant results were seen when compared to the same groups of Root ZX (T=2.86, p=0.005)”. More accurate? If so, not true statement related to table 3?

 Thank you for your comment, it is adjusted in the discussion.

“In the case of using NaOCl as an irrigant, it showed significant results when used with Raypex 6 even without coronal pre-flaring” compared to what? To RootZX? With pre flaring it is less accurate.

 In case of using NaOCL as an irrigant the mean score was significantly higher in Raypex 6 apex locator for coronal pre-flaring (0.464±0.64, p-<0.001)

The results showed that the readings when using Raypex 6 (6 ^th^ generation electronic apex locator) are significantly closer to the actual working length than Root ZX (3 ^rd^ generation electronic apex locator) for the with/without coronal pre-flaring groups. Doesn’t match with the results of table 3.

 Thank you for your comment. Rephrasing was done as follows to make it clearer. The results showed that the readings when using Raypex 6 (6 ^th^ generation electronic apex locator) are significantly closer to the actual working length than Root ZX (3 ^rd^ generation electronic apex locator) for the with/without coronal pre-flaring in CHX irrigant system and in overall comparison when irrigant systems were not considered.
